# TRAF2 Promotes Liver Fibrosis via Regulation of the HIF-1α/GLUT1-Mediated Glycolysis in Hepatic Stellate Cells

**DOI:** 10.7150/ijbs.99682

**Published:** 2025-09-03

**Authors:** Yina Zhang, Siduo Xu, Jiajia Shao, Yining Lu, Lingzhu Zhao, Xue Liang, Jiping Yao, Minwei Li, Yanning Liu, Min Zheng

**Affiliations:** 1 State Key Laboratory for Diagnosis and Treatment of Infectious Diseases, National Clinical Research Center for Infectious Diseases, China-Singapore Belt and Road Joint Laboratory on Infection Research and Drug Development, National Medical Center for Infectious Diseases, Collaborative Innovation Center for Diagnosis and Treatment of Infectious Diseases, The First Affiliated Hospital, College of Medicine, Zhejiang University, Hangzhou, 310003, China.; 2 Yuhang Institute of Medical Science Innovation and Transformation, Hangzhou, 310000, China.

**Keywords:** TRAF2, Liver fibrosis, Hepatic stellate cells (HSCs), Glycolysis, HIF-1α, GLUT1

## Abstract

Tumor necrosis factor receptor-associated factor 2 (TRAF2) is an intracellular aptamer protein with E3 ligase activity and has been reported to be involved in the pathogenesis of hepatitis and liver cancer. However, the specific mechanism for liver fibrosis mediated by TRAF2 is a still-unresolved issue. In this study, we uncovered high TRAF2 expression in activated hepatic stellate cells (HSCs) and fibrotic livers of both human and two mouse liver fibrosis models. TRAF2 in HSCs correlated positively with liver fibrosis and could directly prompt HSC activation, as evidenced by *in vitro* gain-of-function and loss-of-function models. *In vivo*, HSC-specific knockout of TRAF2 could alleviate liver injury and fibrosis in mice. Mechanistically, we demonstrated that TRAF2 in HSCs promoted the increase of hypoxia-inducible factor-1α (HIF-1α) levels by inhibiting von Hippel-Lindau (pVHL)-mediated HIF-1α degradation and inducing HIF-1α translation via activating mTORC1 pathway. Elevated HIF-1α expression predisposed to a rise in its transcriptional target glucose transporter 1 (GLUT1) expression and glycolytic activity in HSCs, eventually developing liver fibrosis. Thus, TRAF2 exerts a significant impact upon activating HSCs and may become a candidate molecule for anti-liver fibrosis therapy.

## Introduction

Liver fibrosis is mainly manifested, whatever the etiology, as the pathological buildup of extracellular matrix (ECM) following continuous liver injury 1-3. Previous studies have confirmed that characterized with transcriptional and epigenetic remodeling, the continuous activation of hepatic stellate cells (HSCs) is thought to be the core event in liver fibrogenesis 2. Despite making up only 5-8% of the total of liver cells, HSCs are typically assumed to be the major cellular sources of myofibroblasts under pathological conditions, which resemble fibroblast-like cells manifesting profibrogenic and ECM-producing properties 2, 4. ECM molecules accumulate and constitute pathological fiber tissue in the space of Disse, culminating in the reconstruction of intrahepatic structure and liver fibrosis 2, 3, 5. Affected by continuous pathological stimulation, liver fibrosis can progress into a more serious pathological outcome and eventually develop into cirrhosis 2. If not treated effectively and promptly, cirrhosis can lead to a series of lethal complications, including portal hypertension-induced esophageal and gastric variceal rupture and hemorrhage, ascites, and infection, liver failure, hepatic encephalopathy and liver cancer 2, 6.

Tumor necrosis factor receptor-associated factors (TRAFs), initially functioned as intracellular adaptor proteins, were responsible for signal transduction of the tumor necrosis factor receptor (TNFR) superfamily 7, 8. TRAF2, a well-studied member of TRAF family, exists in a wide range of tissues and cell types. It is now recognized that TRAF2 concerning multiple cellular processes includes nuclear factor-κB (NF-κB) and mitogen-activated protein kinase (MAPK) cascade pathway, endoplasmic reticulum (ER) stress signaling, autophagy, cellular proliferation, differentiation, and apoptosis 9-15. So far, numerous studies have discussed and determined the function and molecular mechanism of TRAF2 in certain liver diseases, especially hepatitis and liver cancer, nevertheless, the regulatory role and latent mechanism of TRAF2 in liver fibrosis still needs to be elucidated 16-20. Recent findings have revealed that TRAF2 may be involved in the pathogenesis of certain tissue fibrosis-related diseases, such as renal interstitial fibrosis and pancreatic fibrosis 21-23. Besides, the expression of TRAF2 was markedly elevated in fibrotic liver samples from humans in comparison to healthy liver tissues 24. These studies imply a crucial role of TRAF2 in regulating liver fibrosis.

Therefore, we sought to inquire into the profibrotic role of TRAF2 by combination of HSC-specific conditional TRAF2-knockout mice and LX-2 cells, a human HSC line. Our current research found a vital role of TRAF2 in activating HSCs in the context of liver fibrosis. Liver fibrosis in mouse models was alleviated by specifically deleting TRAF2 in HSCs. *In vitro*, TRAF2 knockdown inhibited the levels of a series of markers related to the activation of HSC and fibrosis, whereas these phenotypes were reversed by TRAF2 overexpression. The mechanistic study demonstrated that TRAF2 regulated glycolysis in liver fibrosis via hypoxia-inducible factor 1α (HIF-1α) and its transcriptional target glucose transporter type 1 (GLUT1). Together, our study provides concrete experimental evidence that TRAF2 is a latent promising target for treating liver fibrosis.

## Materials and Methods

### Human liver samples

Liver samples were acquired from the First Affiliated Hospital, Zhejiang University School of Medicine and approved by the Ethics Review and Scientific Investigation Board of the hospital, with all patients giving their informed consent. We collected liver fibrosis/cirrhosis samples from individuals who underwent liver transplantation due to liver failure, of whom 9 cases were diagnosed with hepatitis B virus (HBV)-associated fibrosis/cirrhosis and 6 cases receiving a diagnosis of alcohol-induced liver fibrosis/cirrhosis. In addition, we also collected 10 adjacent normal liver tissue samples from individuals who underwent liver resection for hepatic hemangioma as controls.

### Animal studies

Animal protocols were approved by the Animal Experimental Ethics Committee of the First Affiliated Hospital, Zhejiang University School of Medicine. C57BL/6 male mice that met the requirements for weeks of age and body weight, TRAF2-flox mice (strain no. T007336), lecithin retinol acyl transferase (Lrat)-driven cyclization recombinant enzyme (Cre) mice (strain no. T006205) and albumin (Alb)-Cre mice (strain no. T003814) were purchased from GemPharmatech. The background of all transgenic mouse strains originated from C57BL/6J.

Lrat-Cre mice were crossed with TRAF2 flox/flox (TRAF2^f/f^) mice to generate HSC-specific conditional TRAF2-knockout mice (TRAF2^△HSC^). Similarly, Alb-Cre mice were crossed with TRAF2^f/f^ mice to generate hepatocyte-specific conditional TRAF2-knockout mice (TRAF2^△Alb^). The genotypes of transgenic mice were identified 10 days after birth according to the protocol provided by GemPharmatech. All mice were kept in sterile conditions at a temperature of 22 to 24°C with a 12 h light-12 h dark cycle and allowed ad libitum access to fodder and water.

The liver fibrosis model induced by carbon tetrachloride (CCl_4_; #65805; Adamas-beta, China) in mice was carried out as previously described 25. In brief, male 8 weeks of age C57BL/6 mice weighing 20-25 g were induced by twice-weekly intraperitoneal injections of a 20% solution of CCl_4_ (1 ml/kg) or the equivalent solvent (i.e. olive oil) alone for 8 weeks. Three days after the final CCl_4_ injection, mouse orbital blood collected for the subsequent biochemistry test of liver function was done, then mice were sacrificed and dissected for obtaining the liver. A portion of hepatic right lobe was cut and immersed in 10% buffered formalin for histological examination. The other liver tissues were divided into several small pieces and packed in several freezing tubes, followed by a rapid freezing using liquid nitrogen and a long-term preservation at -80°C until use.

To establish the cholestatic liver fibrosis model in mice, common bile ducts of male 8 weeks of age C57BL/6 mice weighing 20-25 g were ligated, while the control mice received the same operation procedure except ligation. On day 15 after surgery, all mice were sacrificed for harvesting the livers and blood samples, which were further processed as described above.

### Primary liver cells isolation

Nycodenz density gradient centrifugation method was applied for the isolation of primary mouse HSCs as described previously 26. We made slight modifications on the procedure of perfusion. In general, the portal vein was cannulated for a smooth stepwise infusion with solutions containing pronase (#10165921001; Roche, Switzerland) and collagenase IV (C5138; Sigma, USA). Of note, making an incision in the inferior vena cava preceded the digestion of the liver, in order to open an avenue for perfusate escaping from the catheter. Finally, the cells were subjected to Nycodenz density gradient centrifugation for gaining HSCs. A two-step collagenase perfusion method previously described was used as a reference to achieve the isolation of primary mouse hepatocytes 27. Subsequently, a cell strainer with a mesh aperture of 70 µm was selected for hepatocytes filtration. Finally, the hepatocytes were harvested after being centrifuged (Setting: 50 g, 5 min).

### Cell culture and treatment

LX-2 cells and the isolated mouse HSCs were cultured in high-glucose Dulbecco's modified Eagle's medium (DMEM) (Gibco, USA) supplemented with 10% fetal bovine serum (FBS; Gibco, USA) and 1% penicillin/streptomycin (Biosharp, China). Meanwhile, the cells were maintained in an incubator set to a temperature of 37°C and a humidified atmosphere of 95% air and 5% CO2.

All small interfering RNA (siRNA) and plasmid used for the transfection assays were purchased from GenePharma. Gene knockdown mediated by siRNA in LX-2 cells was achieved by the use of Lipofectamine™ RNAiMAX transfection reagent (#13778150; Invitrogen, USA). Plasmid (GenePharma, China) was delivered into LX-2 cells by Lipofectamine 3000 (L3000015; Invitrogen, USA) and P3000™ (L3000015; Invitrogen, USA). After a 4-6 h of co-incubation, the old supernatant was swapped out for fresh medium for additional 48 h incubation. Then, cells were collected and further analyzed. To investigate crosstalk between hepatocytes and HSCs, a medium transfer experiment was utilized in this study. Specifically, the human hepatocyte cell line HepG2 or Huh7 cells were transfected with Ctrl siRNA or TRAF2 siRNA in 6-well plates. After incubation for 4-6 h, the supernatant was replaced with fresh culture medium for another 48 h-incubation. Then, conditioned medium (CM) collected from the hepatocyte cell lines was subsequently loaded into the LX-2-containing wells. Finally, the LX-2 cells were collected and analyzed after 24 h culture. To inhibit GLUT1, LX-2 cells were exposed to 50 nM and 100 nM BAY-876 (HY-100017; MedChemExpress, USA) or 1‰ dimethylsulfoxide (DMSO; D2650; Sigma-Aldrich, USA) for 24 h. To evaluate whether TRAF2 regulated proteasomal degradation of HIF-1α, 10 µM MG132 was selected to add into LX-2 cells silencing TRAF2 or not for a subsequent 6 h-treatment. To assess whether TRAF2 affected HIF-1α levels by regulating the mammalian target of rapamycin (mTOR) Complex 1 (mTORC1) signaling, we utilized 100 nM Rapamycin (HY-10219; MedChemExpress, USA) to treat LX-2 cells for additional 24 h after TRAF2 plasmid transfection for 48 h.

### Lipid droplets (LDs) staining

The procedure of LDs staining was performed as previously described 28. In brief, primary HSCs isolated from normal mice were cultured *in vitro* for 6 h, 3, or 7 days. Cells were formaldehyde-fixed for 10-30 min and labeled with LipidTOX™ Red neutral lipid stain (H34476; Invitrogen, USA) according to the protocol of the manual. After a 30 min-incubation at room temperature, neutral lipid accumulation was detected and imaged by fluorescencemicroscopes.

### Extraction of proteins from the cytoplasm and nucleus

Proteins from the cytoplasm and nucleus were obtained following the guidelines of a nuclear and cytoplasmic protein extraction kit (P0027; Beyotime, China).

### Detection of HIF-1α transcriptional activity

Simply put, LX-2 cells were co-transfected with HIF-1-LUC luciferase reporter (#11520ES03; Yeason, China) with or without TRAF2 plasmid for 48 h. The Renilla luciferase reporter plasmid served as a transfection control. Afterwards, the luminescence signal in these cells was determined following the instructions provided by a dual luciferase reporter assay kit (RG029, Beyotime, China).

### Glucose uptake assay

LX-2 cells cultured on a glass-bottom Petri dish were rinsed with phosphate buffer saline (PBS) after a 48 h-transfection with vehicle or TRAF2 siRNA. Then, glucose-free DMEM (G4528; Servicebio, China) containing the fluorescent glucose analogue 2-(N-(7-nitrobenzen-2-oxa-1, 3-diazol-4-yl)amino)-2-deoxyglucose (2-NBDG, 100 µM; HY-116215; MCE, USA) was added to cells for a 30 min-incubation. Afterwards, the cells rinsed thrice with PBS were further processed. The mean fluorescence intensity (MFI) of 2-NBDG was measured via flow cytometry, while images were photographed and obtained with a Zeiss laser scanning confocal microscope.

### Detection of glucose and lactate content

The testing of glucose and lactate levels in the cell supernatant were performed with a glucose assay kit (G264; Dojindo, China) and a lactate assay kit (L256; Dojindo, China), respectively, according to the guidelines provided by the manufacturer.

### Cell proliferation assay by immunodetection of 5-ethynyl-2'-deoxyuridine (EdU) incorporation

The quantitative evaluation of cell proliferation can be achieved by measuring deoxyribonucleic acid (DNA) synthesis during the S phase of the cell cycle using EdU, a synthetic analog of thymidine. Briefly, TRAF2 siRNA was added to LX-2 cells to achieve the effect of knocking down TRAF2 in the cells, and the control was set simultaneously using Ctrl siRNA to treat LX-2 cells. Then, following the guidelines from a EdU-488 cell proliferation assay kit (CX002; Epizyme, China), 10 µM EdU was administered into the cells. After a 2 h-treatment, LX-2 cells were rinsed using PBS and fixed using 4% paraformaldehyde, with a subsequent permeabilization using 0.25% Triton X-100. EdU was then labeled with the fluorescent dye Alexa Fluor 488 via a click reaction. LX-2 cells were washed and then exposed to 4,6-diamidino-2-phenylindole (DAPI; G1012; Servicebio, China) in a dark environment for nuclear staining. The percentage of EdU positive cells was visualized and photographed with a Zeiss laser scanning confocal microscopy.

### Co-immunoprecipitation (Co-IP)

Co-IP assay was conducted according to the manual from a classic Co-IP assay kit (YJ201; Epizyme, China). In short, lysis/rinsing buffer supplemented with protease inhibitor cocktail tablets (#04693132001; Roche, Switzerland) and phosphatase inhibitor tablets (#4906837001; Roche, Switzerland) was used to lyse cells. After centrifugation, the cell lysates were divided into two layers, with the upper liquid containing cell proteins. 60 µL of the supernatant was retained for immunoblotting analysis, while the rest underwent 1 h-incubation with the indicated antibodies, with another 1 h-incubation with protein A/G-conjugated agarose beads. Two procedures of incubation were completely carried out at the room temperature. The antigen-antibody-magnetic bead complex were rinsed at least 3 times with the lysis/rinsing buffer. Finally, the protein was eluted from the beads for carrying out the subsequent immunoblotting procedure.

### Immunoblotting analysis

Immunoblotting was performed as previously described 27. In brief, preparing RIPA lysis buffer containing inhibitors against protease and phosphatase lysed cells or tissue samples to gain total protein. The extracted protein was further processed for quantification and subsequently boiled with 1 × SDS loading buffer. The appropriate SDS-PAGE precast gel was selected for applying electrophoresis method to separate different protein according to their different molecular weight. Then the protein entering the gel was transferred to a PVDF membrane (IPVH00010; Millipore, USA) with the help of a rapid transfer system. The membrane was sealed with 5% bovine serum albumin (BSA; GC305010; Servicebio, China) dissolved in TBST for 1 h, followed by an overnight incubation with the primary antibodies of interest at 4°C. Afterwards, the membrane was rinsed 3 times using TBST and then kept in TBST containing the corresponding secondary antibodies at room temperature for 1 h. Finally, bands were visualized by enhanced chemiluminescence (ECL) system. β-actin or GAPDH was referred as an internal control. The information of all antibodies applied in immunoblotting are recorded in **Supplementary Table 1**.

### Quantitative reverse-transcription polymerase chain reaction (qRT-PCR) assays

An RNA extraction kit (RK30120; Abclonal, China) was adopt for total RNA extraction. Subsequently, a RT reaction was conducted for cDNA synthesis by virtue of a RT reagent Kit (RR047A; Takara, Japan). qRT-PCR was performed utilizing TB Green® Premix Ex Taq™ II (Tli RNaseH Plus) (RR420A; Takara, Japan). Genes of interest were normalized to β-actin. All PCR primer sequences applied in this study are recorded in **Supplementary Table 2**.

### Histology, immunohistochemistry and immunofluorescence

Paraffin sections containing mouse liver tissues stained with hematoxylin and eosin (H&E) and sirius red were prepared to visualize inflammation and liver fibrosis. The procedures of immunohistochemical and immunofluorescence staining were as described previously 29, 30. For immunohistochemical staining, a series of experiments conducted on paraffin-embedded sections included deparaffinage, hydration, inactivation of endogenous peroxidase, serum blocking, and incubation of primary and secondary antibodies. For immunofluorescence staining, paraffin-embedded liver specimens or cells were processed to detect the expression of interested molecules or observe co-localization. The nuclei were labeled using DAPI. Finally, all histological staining results were viewed under a microscope (Olympus BX53, Japan). A detailed record of antibodies applied in immunohistochemistry and immunofluorescence analyses is made in **Supplementary Table 1**.

### Statistical analysis

The results were statistically processed using GraphPad Prism 9.0 software and expressed as the means ± SD. Comparison between two groups was examined by Student's t-test, while multiple comparisons were examined by one-way ANOVA. Generally, *P* < 0.05 was considered statistically significant.

## Results

### TRAF2 expression is upregulated in human and mouse liver fibrosis

**Figure 1:**
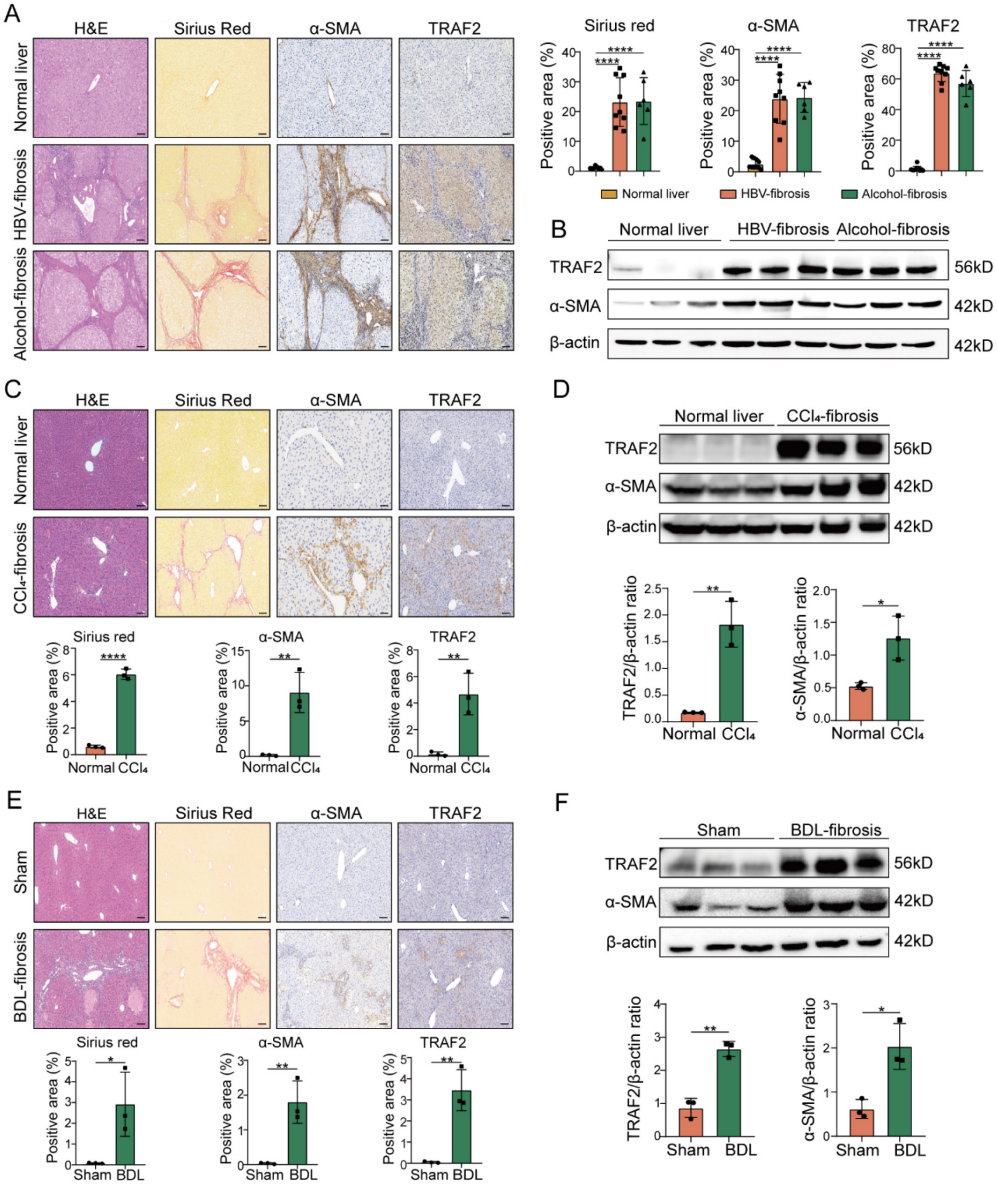
** High TRAF2 abundance was observed in fibrotic livers from human and two mouse liver fibrosis models.** (A) Liver samples obtained from individuals with HBV-related (n = 9) or alcohol-related (n = 6) liver fibrosis underwent H&E, sirius red and immunohistochemistry staining for α-SMA and TRAF2 after paraffin embedding and section. Tissues adjacent to hepatic hemangioma were employed as controls (n = 10). Image Pro Plus software was applied for the measurement of positive staining regions. Scale bars = 50 µm. (B) Protein levels of TRAF2 and α-SMA in clinical samples from HBV or alcohol-related liver fibrosis were detected by immunoblot assay, and hepatic hemangioma-adjacent normal tissues were employed as controls. (C) Liver tissues from mice subjected to olive oil or CCl_4_ after paraffin embedding and section underwent H&E, sirius red and immunohistochemical staining for α-SMA and TRAF2 and relative quantitative results of the regions of positive staining. Scale bars = 50 µm. (D) Protein levels of TRAF2 and α-SMA in the livers from vehicle or CCl_4_ injected-mice were probed using immunoblotting. (E) Liver sections from mice subject to sham operation or BDL underwent H&E, sirius red and immunohistochemical staining for α-SMA and TRAF2 and quantification of the regions of positive staining. Scale bars = 50 µm. (F) Hepatic expression of TRAF2 and α-SMA proteins in mice suffering sham operation or BDL was detected using immunoblotting. All data are represented as the means ± SD. *, *P*<0.05; **, *P*<0.01; ****, *P*<0.0001. **Abbreviations**: H&E: Hematoxylin and eosin; α-SMA: α-smooth muscle actin; TRAF2: Tumor necrosis factor receptor-associated factor 2; HBV: Hepatitis B virus; CCl_4_: Carbon tetrachloride; BDL: Bile duct ligation.

To investigate the correlation between TRAF2 expression and liver fibrosis, we initially evaluated its level in surgical specimens that were diagnosed with HBV-induced fibrosis or alcohol-induced fibrosis. We found that elevated TRAF2 expression in HBV-infected, and alcohol-induced human fibrotic livers compared to non-fibrotic controls as demonstrated by immunohistochemistry and immunoblot assay, which was accompanied by an enhancive protein level of α-smooth muscle actin (α-SMA), a marker of activated HSCs (**Figure 1A and 1B**). Subsequently, we examined the TRAF2 expression profile in two well-established rodent models, in which CCl_4_ induces liver fibrosis in mice by damaging liver cells, and bile duct ligation (BDL) induces liver fibrosis in mice by cholestasis. Likewise, immunohistochemistry analysis using liver tissue sections from murine fibrotic models subjected to CCl_4_ or BDL also demonstrated a notable rise in TRAF2 positive areas in contrast to the normal controls (**Figure 1C and 1E**). Moreover, whole-liver homogenates of protein levels of TRAF2 were notably elevated in murine fibrotic livers than those of the control group (**Figure 1D and 1F**) as demonstrated by immunoblotting. The data seem to demonstrate that high abundance of TRAF2 exists in fibrotic livers during liver fibrosis.

### Upregulation of TRAF2 in HSCs and hepatocytes is linked to liver fibrosis

**Figure 2:**
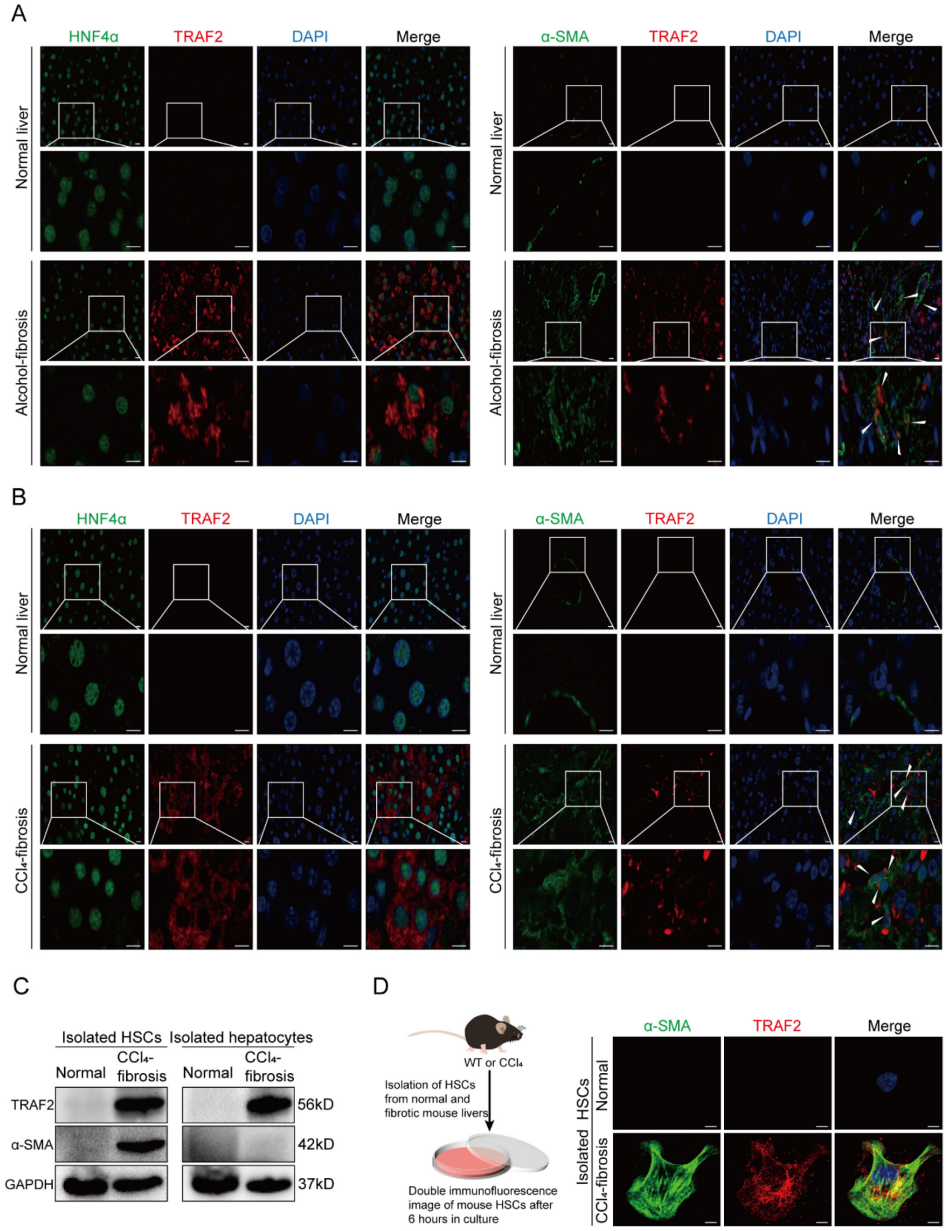
** Increased TRAF2 expression was basically localized in HSCs and hepatocytes in fibrotic livers of humans and mice.** (A) Normal and fibrotic liver tissues of humans were stained with an anti-HNF4α or anti-α-SMA antibody (green) for labeling hepatocytes and HSCs, respectively. Fluorescent TRAF2 was shown in red and the cell nucleus stained with DAPI was shown in blue. Scale bars = 10 µm. (B) Vehicle or CCl_4_-induced liver tissues of mice were stained with an anti-HNF4α or anti-α-SMA antibody (green) for labeling hepatocytes and HSCs, respectively. Fluorescent TRAF2 was shown in red and the cell nucleus stained with DAPI was shown in blue. Scale bars = 10 µm. (C) Liver tissues from mice receiving olive oil or CCl_4_ underwent perfusion and enzyme digestion for isolating primary HSCs and hepatocytes, followed by the confirmation of TRAF2 and α-SMA proteins in these two kinds of cells by immunoblotting analyses. (D) Double immunofluorescence was done on the primary HSCs isolated from mice receiving olive oil or CCl_4_. Fluorescent α-SMA was displayed in green, fluorescent TRAF2 was shown in red and the cell nucleus stained with DAPI was shown in blue. Scale bars = 5 µm. **Abbreviations**: HNF4α: Hepatocyte nuclear factor 4α; α-SMA: α-smooth muscle actin; TRAF2: Tumor necrosis factor receptor-associated factor 2; DAPI: 4,6-diamidino-2-phenylindole; CCl_4_: Carbon tetrachloride; HSC: Hepatic stellate cell; WT: Wild type.

To further define in which liver cell types TRAF2 expression was significantly upregulated during liver fibrosis, we first performed immunofluorescence double staining experiments on human normal and cirrhotic livers to study the co-localization of TRAF2 with hepatocyte nuclear factor 4α (HNF4α), α-SMA, cluster of differentiation (CD) 68, CD31 and cytokeratin (CK) 19, which represent cell markers of hepatocytes, activated HSCs, Kupffer cells, endothelial cells and biliary tract cells, respectively. The red areas were represented by TRAF2 positive staining and the green areas were represented by cell marker positive staining. Surprisingly, among the cell types evaluated, parenchymal areas and cells exhibiting a morphology resembling that of myofibroblasts in fibrotic tissues displayed abundant expression of TRAF2, suggesting an increase in TRAF2 expression specifically in hepatocytes and HSCs during liver fibrosis (**Figure 2A**), with no significant change in three other kinds of liver cells (**Figure S1A, S1B and S1C**). Consistently, immunofluorescence double staining analyses showed enhanced TRAF2 expression mainly in hepatocytes and HSCs of fibrotic liver sections from mice subjected to CCl_4_ (**Figure 2B; Figure S2A, S2B and S2C**). Moreover, we successfully isolated primary HSCs from normal mice and identified that freshly isolated HSCs were rich in LDs by LipidTOX™ neutral lipid stain and the spontaneous activation of these isolated HSCs cultured on dishes was accompanied by the rapid loss of intracellular LDs (**Figure S3**). Next, we further explored the differential expression of TRAF2 between primary HSCs isolated from normal or CCl_4_-treated mice. The results confirmed that primary HSCs from mice which were treated with CCl_4_ exhibited increased expression of TRAF2, compared with the controls (**Figure 2C and 2D; Figure S4A**). Similarly, we also unmasked a higher level of TRAF2 in hepatocytes isolated from mouse fibrotic livers than the controls (**Figure 2C; Figure S4B**).

Interestingly, we observed that as compared with TRAF2^f/f^ mice, the CCl_4_ and BDL model caused less collagen deposition in hepatocyte-specific conditional TRAF2-knockout (TRAF2^ΔAlb^) mice according to the results of sirius red (**Figure S5A and S5B**). It is previously reported that hepatocytes promote liver fibrosis through communication with HSCs during the process of chronic liver injury 27, 31-34. We therefore utilized a medium transfer experiment and attempted to explore the possible indirect effect of TRAF2 in hepatocytes on HSC activation (**Figure S6A**). We found that knockdown of TRAF2 in hepatocytes dramatically impacted HSC activation, which was mainly manifested as a significant reduction in the protein and mRNA levels of TRAF2 and HSC activation markers in TRAF2 knockdown hepatocyte-derived CM-cultured HSCs, as compared to that of controls (**Figure S6B, S6C and S6D**). In fact, as the central hallmark of liver fibrosis, HSCs are generally recognized as the predominant contributor responsible for ECM deposition. It has been reported that activated HSCs express much higher levels of fibrosis-related molecules than hepatocytes 32, 35. By isolating hepatocytes and activated HSCs from fibrotic liver caused by CCl_4_ and comparing some liver fibrosis-related indicators detected in these isolated cells, we observed higher mRNA levels of liver fibrosis-related molecules in HSCs than hepatocytes (**Figure S7**). Taken together, these observations signify that high TRAF2 expression in HSCs may directly affect the onset and progression of liver fibrosis, whereas TRAF2 upregulation in hepatocytes is more likely to play an indirect role to contribute to HSC activation and liver fibrosis via cell-to-cell communication between hepatocytes and HSCs.

### HSC-specific ablation of TRAF2 mitigates CCl_4_- and BDL-induced liver fibrosis in mice

Our core work aims to clarify the HSC-specific function of TRAF2 in liver fibrosis *in vivo*, thus, we established HSC-specific conditional TRAF2-knockout mice (TRAF2^△HSC^) by hybridization of Lrat-driven Cre mouse line and TRAF2-flox mouse line. Littermates (TRAF2^f/f^ mice) were used as controls. Genotype identification of TRAF2^ΔHSC^ and TRAF2^f/f^ transgenic mice was conducted by direct PCR (**Figure S8A**). We confirmed that TRAF2 was specifically abrogated in HSCs isolated from livers of TRAF2^ΔHSC^ transgenic mice, rather than TRAF2^f/f^ transgenic mice (**Figure S8B**). Simultaneously, TRAF2 deletion in HSCs was proved to be unable to influence the endogenous expression of Lrat (**Figure S8B**). Furthermore, dual immunofluorescence conducted on liver tissue sections from TRAF2^ΔHSC^ and TRAF2^f/f^ transgenic mice uncovered a complete overlap of the fluorescent signals of Cre and desmin (a marker of HSCs) in TRAF2^ΔHSC^ mice (**Figure S8C**). These results indicated the successful generation of HSC-specific conditional TRAF2-knockout mice.

**Figure 3:**
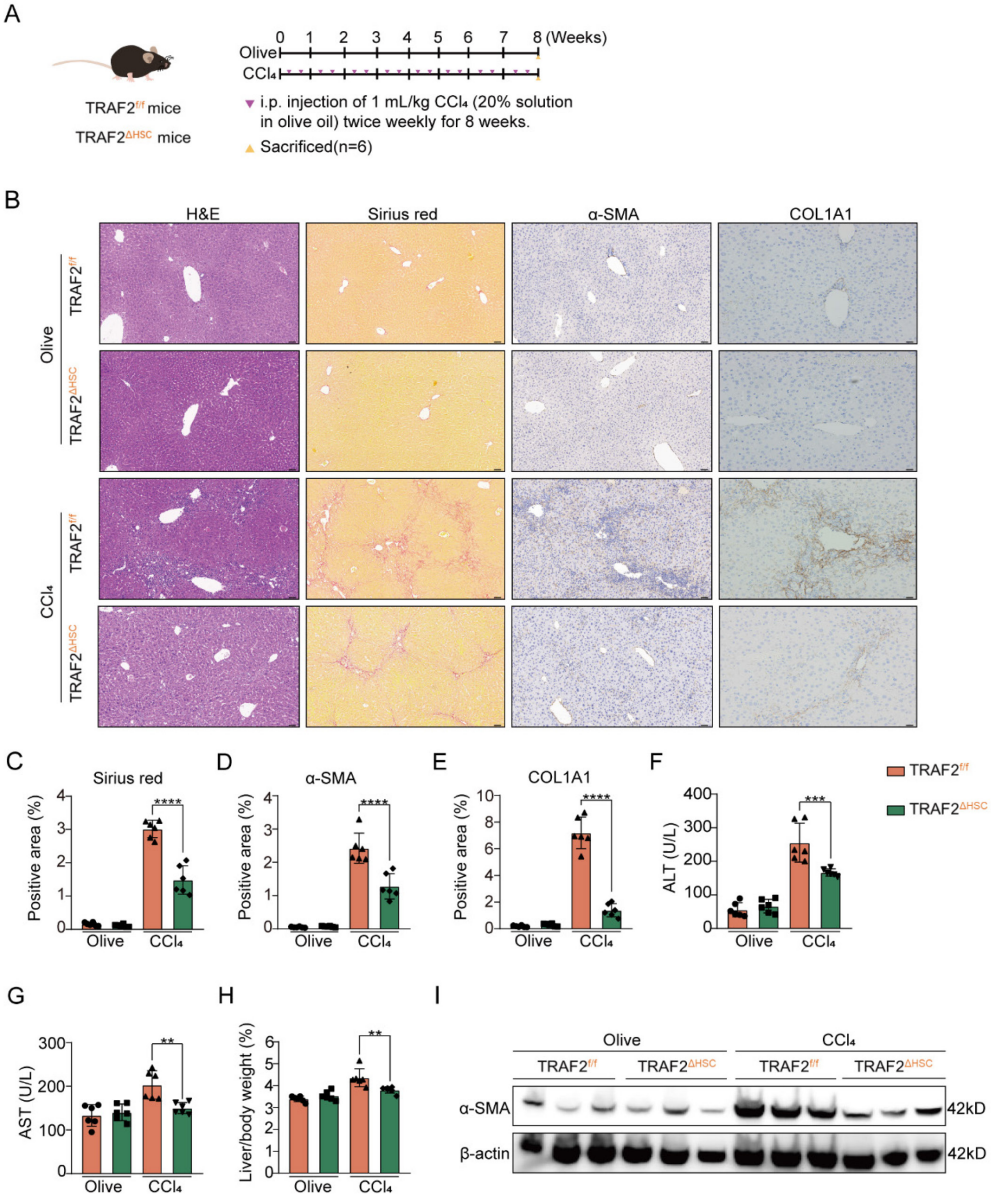
** HSC-specific ablation of TRAF2 protected against liver fibrosis in mice after CCl_4_ treatment.** (A) Schematic illustration of TRAF2^f/f^ and TRAF2^△HSC^ mice receiving twice-weekly intraperitoneal injections of olive oil or CCl_4_ for 8 weeks. (B) Liver tissues from TRAF2^f/f^ and TRAF2^△HSC^ mice after paraffin embedding and section underwent H&E, sirius red as well as immunohistochemical staining for α-SMA and COL1A1. Scale bars = 50 µm. (C-E) Relative quantitative results of the regions of sirius red, α-SMA and COL1A1 positive staining. (F-G) ALT and AST were evaluated as indicators of liver function. (H) Statistic analysis showed the liver/body weight ratio. (I) Hepatic α-SMA expression in transgenic mice receiving vehicle or CCl_4_ was detected using immunoblotting. All data are represented as the means ± SD. **, *P*<0.01; ***, *P*<0.001; ****, *P*<0.0001. **Abbreviations**: H&E: Hematoxylin and eosin; α-SMA: α-smooth muscle actin; COL1A1: Collagen type 1; TRAF2: Tumor necrosis factor receptor-associated factor 2; CCl_4_: Carbon tetrachloride; HSC: Hepatic stellate cell; ALT: Alanine aminotransferase; AST: Aspartate aminotransferase.

Then, we studied the effect of TRAF2 knockout in HSCs on CCl_4_- and BDL-induced mouse liver fibrosis. As illustrated in **Figure 3A**, TRAF2^ΔHSC^ and TRAF2^f/f^ transgenic mice were exposed to either olive oil or CCl_4_ twice weekly for 8 weeks. Our preliminary experimental evidence based on H&E and sirius red analysis indicated that, TRAF2^f/f^ transgenic mice subjected to CCl_4_ predisposed to liver injury and collagen deposition compared with TRAF2^ΔHSC^ transgenic mice receiving CCl_4_ (**Figure 3B and 3C**). Consistently, deficiency of TRAF2 in HSCs caused lower levels of α-SMA and collagen type 1 (COL1A1) as assessed by immunohistochemistry (**Figure 3B, 3D and 3E**;* P* values all < 0.0001). The alleviation of fibrosis observed in TRAF2^ΔHSC^ transgenic mice subjected to CCl_4_ might be the result of remission of liver damage. As anticipated, two serum liver function index, alanine aminotransferase (ALT) and aspartate aminotransferase (AST), displayed a definite reduction in TRAF2^ΔHSC^ mice (**Figure 3F and 3G**). In the CCl_4_ model, we found that the deletion of TRAF2 in HSCs appeared to have markedly reduced liver/body weight ratio in transgenic mice than TRAF2^f/f^ mice (**Figure 3H**; *P* value < 0.01), whereas their body weight showed no significant difference during the 8 weeks of CCl_4_ treatment (**Figure S9A and S9B**). In addition, as detected by the immunoblotting result, TRAF2^ΔHSC^ mice exhibited a significant diminishment of α-SMA protein after treating CCl_4_ in comparison to TRAF2^f/f^ mice (**Figure 3I**), in complete consistency with the result of quantitative analysis of positive areas by α-SMA immunohistochemical staining.

**Figure 4:**
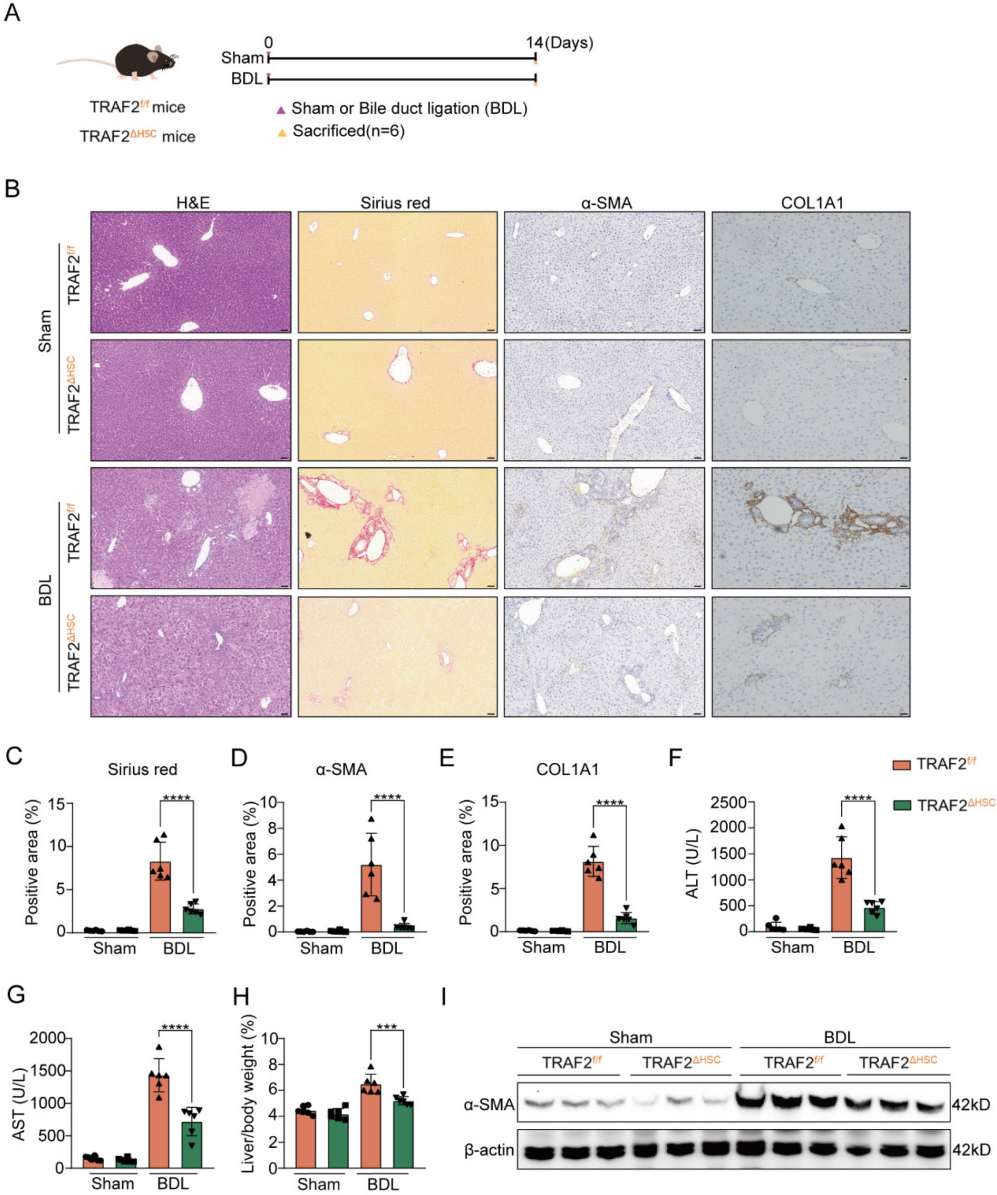
** HSC-specific ablation of TRAF2 protected against liver fibrosis in mice after BDL treatment.** (A) Schematic diagram of TRAF2^f/f^ and TRAF2^△HSC^ mice receiving sham operation or BDL. (B) Livers from transgenic mice after paraffin embedding and section underwent H&E, sirius red and immunohistochemical staining for α-SMA and COL1A1. Scale bars = 50 µm. (C-E) Relative quantitative results of the regions of sirius red, α-SMA and COL1A1 positive staining. (F-G) ALT and AST were evaluated as indicators of liver function. (H) Statistic analysis showed the liver/body weight ratio. (I) Hepatic α-SMA expression in transgenic mice receiving sham operation or BDL was detected using immunoblotting. All data are represented as the means ± SD. ***, *P*<0.001; ****, *P*<0.0001. **Abbreviations**: H&E: Hematoxylin and eosin; α-SMA: α-smooth muscle actin; COL1A1: Collagen type 1; TRAF2: Tumor necrosis factor receptor-associated factor 2; BDL: Bile duct ligation; HSC: Hepatic stellate cell; ALT: Alanine aminotransferase; AST: Aspartate aminotransferase.

We also selected the mouse BDL model to further test HSC-specific function of TRAF2 in liver fibrosis. As illustrated in **Figure 4A**, both TRAF2^f/f^ and TRAF2^△HSC^ mice were split into two groups, respectively: sham-operation control group (Sham group) and BDL-operation liver fibrosis group (BDL group). After 14 days post-operation, mice were sacrificed for acquirement of liver tissues and blood samples. Preliminary evidence based on the results of H&E, sirius red and immunohistochemistry reflected the deletion of TRAF2 in HSCs significantly reduced ECM, α-SMA and COL1A1 levels compared to TRAF2^f/f^ mice in BDL models (**Figure 4B, 4C, 4D and 4E**;* P* values all < 0.0001). Upregulation of indexes of hepatic function was repressed in BDL-operated TRAF2^△HSC^ mice in comparison to TRAF2^f/f^ mice receiving BDL (**Figure 4F and 4G**;* P* values all < 0.0001). TRAF2^△HSC^ mice had reduced liver/body weight ratio (**Figure 4H**; *P* value < 0.001) after BDL surgery and there was no obvious body weight change for TRAF2^f/f^ and TRAF2^△HSC^ group under the same conditions before the mice were sacrificed (**Figure S9C**). Additionally, TRAF2^△HSC^ mice showed a more notable reduction in α-SMA protein expression in whole-liver homogenates following BDL surgery compared to TRAF2^f/f^ mice, in concordance with histopathology of it (**Figure 4I**). Taken together, our observations confirmed that the ablation of TRAF2 in HSCs caused reduced susceptibility to liver fibrosis in mice to the induction of CCl_4_ and BDL models.

### TRAF2 promotes HSC activation and profibrogenic phenotype *in vitro*

**Figure 5:**
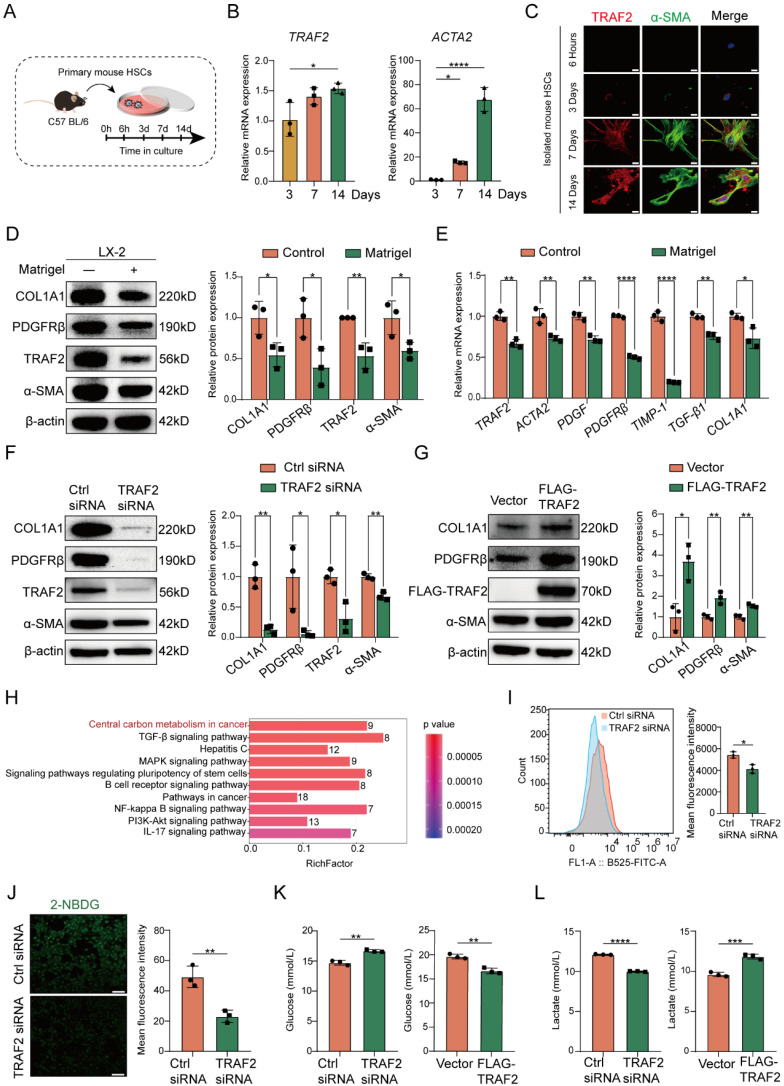
** TRAF2 promoted HSC activation, profibrogenic phenotype and glycolysis *in vitro*.** (A) Schematic illustration of primary mouse HSCs cultured at specified time intervals. (B) Livers from normal mice underwent perfusion and enzyme digestion for isolating primary HSCs and culturing these cells *in vitro* for 3, 7, 14 days, and then determination of the transcriptional levels of *TRAF2* and *ACTA2* was done on the cultured HSCs. (C) Comparison of double immunofluorescence results of primary mouse HSCs cultured for 6 h, 3, 7 and 14 days. Fluorescent α-SMA was displayed in green and fluorescent TRAF2 was shown in red. Scale bars = 10 µm. (D) TRAF2, COL1A1, PDGFRβ and α-SMA levels in LX-2 cells cultured on Matrigel were detected using immunoblotting. (E) Transcript levels of *TRAF2* and indicated fibrosis-related markers in LX-2 cells cultured on Matrigel. (F) Immunoblotting analyses of TRAF2, COL1A1, PDGFRβ and α-SMA protein levels in LX-2 cells with TRAF2 inhibition or not. (G) Immunoblotting analyses of TRAF2, COL1A1, PDGFRβ and α-SMA expression in LX-2 cells with TRAF2 overexpression or not. (H) KEGG pathway enrichment analysis based on the identified proteins which were differentially expressed in LX-2 cells. (I) Flow cytometry analyses of the cell uptake of 2-NBDG. (J) The cell uptake of 2-NBDG was determined using immunofluorescence. (K) The cell supernatants collected were processed to assess glucose concentration. (L) The cell supernatants collected were processed to examine lactate concentration. All data are represented as the means ± SD. *, P<0.05; **, P<0.01; ***, P<0.001; ****, P<0.0001. **Abbreviations**: HSC: Hepatic stellate cell; TRAF2: Tumor necrosis factor receptor-associated factor 2; α-SMA: α-smooth muscle actin; COL1A1: Collagen type 1; PDGFRβ: Platelet-derived growth factor receptor β; KEGG: Kyoto Encyclopedia of Genes and Genomes; 2-NBDG: 2-(N-(7-nitrobenzen-2-oxa-1, 3- diazol-4-yl) amino)-2-deoxyglucose.

Next, we sought to evaluate the impact of TRAF2 on HSC activation. It is known that the isolated HSCs are sensitized to gradually activation within 2 weeks when exposed to fibrogenic stimuli during culture on plastic surfaces 26. Therefore, we firstly cultured isolated mouse HSCs *in vitro* and traced TRAF2 expression during spontaneous HSC activation and harvested mouse HSCs after 3, 7, or 14 days in culture (**Figure 5A**). We observed significantly higher *TRAF2* mRNA levels in mouse HSCs on day 14, accompanied by a rise in *ACTA2* mRNA expression during HSC activation in comparison to day 3 post-isolation (**Figure 5B**). Immunofluorescence double staining also indicated a notable rise in TRAF2 expression in culture-activated primary HSCs (**Figure 5C**). It is worth noting that TRAF2 expression was almost undetectable when primary mouse HSCs were in a quiescent-like state at an early stage. It has been reported that activated HSCs cultured on Matrigel coated plates can be reverted to the deactivation 32. Nowadays, LX-2 cell line has been a commonly accepted tool in the field of liver fibrosis research. Many studies have found that LX-2 retained the key properties of HSC signaling, retinol metabolism and fibrosis formation, such as the expression of α-SMA, platelet-derived growth factor receptor β (PDGFRβ), etc. It also secreted pro-collagen and many kinds of matrix metalloproteinases, all of which were key features of activated HSCs 36. Therefore, we cultured LX-2 cells with critical properties of activated HSCs on Matrigel in order to induce their inactivation. We found that LX-2 cells deactivated by Matrigel not only considerably diminished the protein and mRNA levels of molecules associated with HSC activation and fibrosis, but also suppressed TRAF2 protein and mRNA expression (**Figure 5D and 5E**).

Next, we evaluated the effects of ablation of TRAF2 on morphological changes of primary mouse HSCs. We isolated primary HSCs from TRAF2^ΔHSC^ and TRAF2^f/f^ transgenic mice and cultured these isolated primary cells on dishes for inducing their activation. As shown in **Figure S10**, we found that primary cells adhered with an oblate morphology and abundant LDs on the plastic surface of cell culture plates at 6 h. After 3 days in culture, primary mouse HSCs isolated from TRAF2^f/f^ transgenic mice began to exhibit fine cellular processes branching out from their body, with an expanded and flattened shape, indicating a signal of HSC activation. These activated cells became larger and underwent typical myofibroblastlike morphological changes on day 4 in culture. However, ablation of TRAF2 blocked the spontaneous activation process of primary mouse HSCs induced by plastic cell culture dishes. On day 4 in culture, those primary HSCs from TRAF2^ΔHSC^ transgenic mice closely resembled quiescent HSCs in their appearance. To further verify a direct and substantial role of TRAF2 in HSC activation, we first selected TRAF2 siRNA to transfect LX-2 cells for the suppression of TRAF2 expression. Subsequently, there was a remarkable reduction in TRAF2 expression, as well as the protein and transcription levels of HSC activation and fibrosis-related markers following silencing TRAF2 in LX-2 cells, compared with the controls (**Figure 5F; Figure S11A**), while overexpression of TRAF2 led to the reverse effect (**Figure 5G; Figure S11B**). The above results proved that TRAF2 promoted liver fibrosis via serving as a direct regulator in HSC activation.

### TRAF2 promotes HSC glycolysis

In order to gain a deeper insight into how TRAF2 contributed to liver fibrosis, we performed a proteomic analysis with samples from Ctrl siRNA or TRAF2 siRNA-transfected LX-2 cells following 48 h of incubation. Herein, a TMT/iTRAQ quantitative labeling proteomics approach was utilized for screening differentially expressed proteins. To determine the predominant molecular event in TRAF2-silencing LX-2 cells, a Kyoto Encyclopedia of Genes and Genomes (KEGG) enrichment analysis based on differentially expressed proteins was conducted (**Figure 5H**). The results reflected significant intergroup variations in the central carbon metabolism (CCM), Transforming growth factor beta (TGF-β) pathway, MAPK pathway, NF-κB pathway and phosphatidylinositol 3-kinase (PI3K)/protein kinase B (PKB/AKT) pathway, which were all known to be closely linked to inflammation and liver fibrosis. We observed that the most obvious intergroup alteration was in CCM according to the KEGG analysis (**Figure 5H**), putting forward the hypothesis that TRAF2 might influence HSC activation via regulating glucose metabolism. Generally, CCM reprogramming is crucial for activating HSCs, as it upregulates glycolysis responses in HSCs to satisfy its energy demands for transforming into a proliferative and profibrotic phenotype 37.

Next, we tested whether TRAF2 promoted glycolysis to drive HSC activation. 2-NBDG, a fluorescently labeled 2-deoxyglucose analogue, is often selected for tracing cellular glucose metabolism. We monitored the uptake of 2-NBDG by LX-2 and found that knockdown of TRAF2 resulted in lower fluorescence intensity of 2-NBDG compared with the Ctrl siRNA group (**Figure 5I and 5J**). Previous studies have shown that activated HSCs enhanced glucose utilization compared with their quiescent counterparts 38, 39. Our experiment further supported the fact by culturing isolated primary mouse HSCs *in vitro* which underwent spontaneous activation and determining the glucose consumption in their cell supernatant (**Figure S12**). Subsequently, we utilized siRNA or plasmid to interfere with TRAF2 expression in LX-2 cells, and then conducted a quantitative analysis of the glucose and lactate levels in the cell supernatant. As demonstrated in **Figure 5K and 5L**, the glucose uptake and the lactate generation displayed a notable suppression in the cell supernatant of the TRAF2 siRNA group compared with the Ctrl siRNA group. On the contrary, TRAF2 overexpression markedly elevated the glucose uptake and the lactate generation (**Figure 5K and 5L**). These results unmask that TRAF2 may become a new and vital glycolytic activator in HSCs.

### TRAF2 promotes HSC activation via targeting HIF-1α-GLUT1

**Figure 6:**
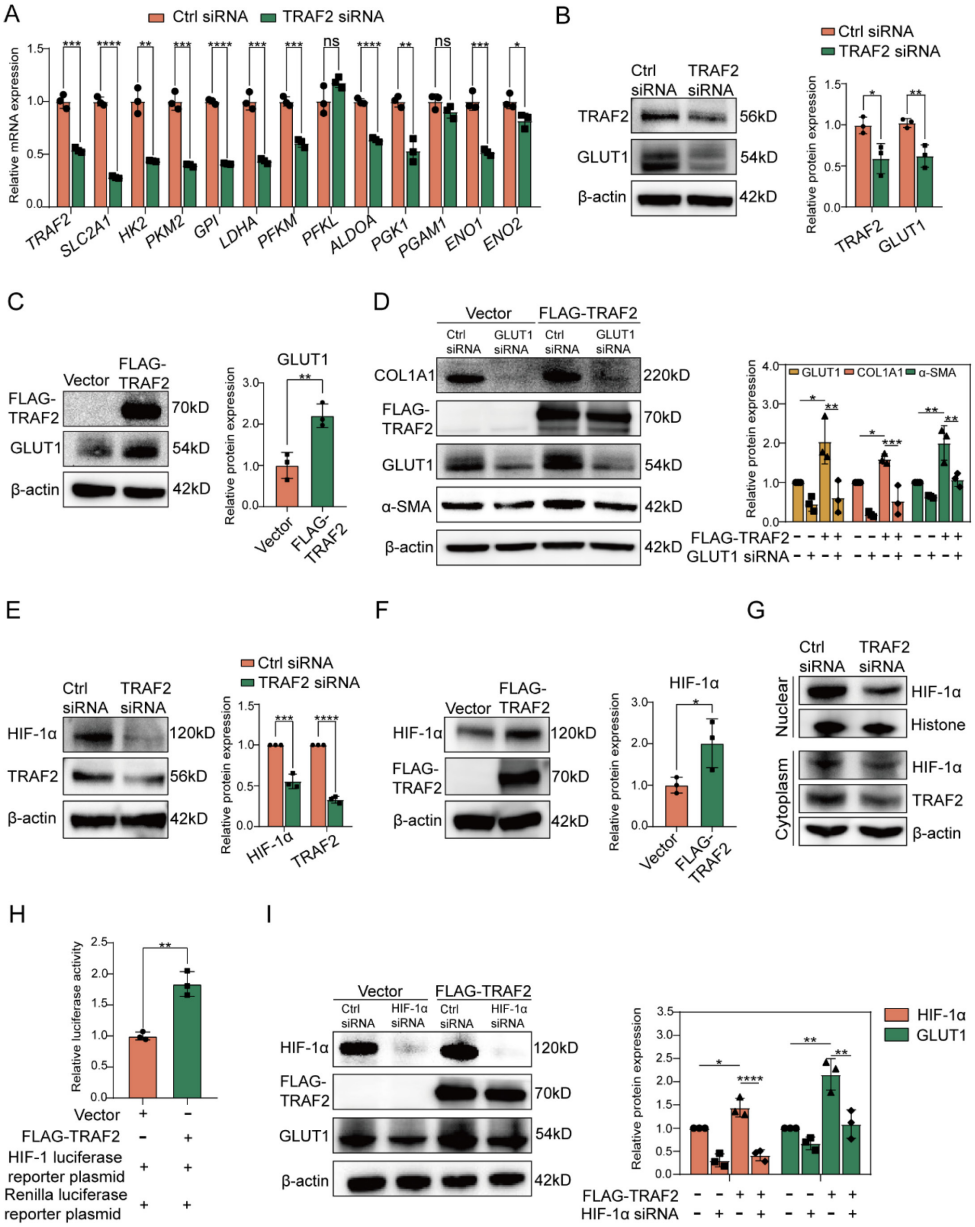
** TRAF2 activated HIF-1α-GLUT1 axis to induce HSC activation and profibrogenic phenotype.** (A) Determination of transcript levels of glycolytic genes. (B) Immunoblotting analyses of protein levels of TRAF2 and GLUT1 after silencing TRAF2 in LX-2 cells. (C) Immunoblotting analyses of TRAF2 and GLUT1 expression in LX-2 cells overexpressing TRAF2 or not. (D) GLUT1 siRNA blocked elevated expression of COL1A1 and α-SMA caused by TRAF2 overexpression. (E) Immunoblotting analyses showed protein levels of TRAF2 and HIF-1α when vehicle or TRAF2 siRNA was administered into LX-2 cells. (F) Immunoblotting analyses of TRAF2 and HIF-1α protein levels in LX-2 cells overexpressing TRAF2 or not. (G) Immunoblotting analyses of nuclear and cytoplasm fraction of HIF-1α after TRAF2 knockdown. (H) LX-2 cells transfected with vector or TRAF2 plasmid for 48 h were collected for determining the transcriptional activity of HIF-1α. (I) Elevated protein level of GLUT1 by TRAF2 overexpression was reversed by HIF-1α siRNA. All data are represented as the means ± SD. *, P<0.05; **, P<0.01; ***, P<0.001; ****, P<0.0001. **Abbreviations**: TRAF2: Tumor necrosis factor receptor-associated factor 2; SLC2A1: Solute carrier family 2 member 1; HK2: Hexokinase 2; PKM2: Phosphofructokinase muscle isoform; GPI: Glucose-6-phosphate isomerase; LDHA: Lactate dehydrogenase A; PFKM: Phosphofructokinase muscle isoform; PFKL: Phosphofructokinase liver isoform; ALDOA: Fructose-bisphosphate aldolase A; PGK1: Phosphoglycerate kinase 1; PGAM1: Phosphoglycerate mutase 1; ENO1: Enolase 1; ENO2: Enolase 2; GLUT1: Glucose transporter 1; α-SMA: α-smooth muscle actin; COL1A1: Collagen type 1; HIF-1α: Hypoxia-inducible factor-1α.

To gain further insight into the association between TRAF2 expression and glycolysis in LX-2 cells, we performed RT-qPCR validation of several key glycolysis-related molecules or rate-limiting enzymes. We found that siRNA to TRAF2 reduced the mRNA levels of most glycolysis-related molecules compared to Ctrl siRNA in LX2 cells (**Figure 6A**). Surprisingly, among the mRNA level evaluated, the downregulation of GLUT1, encoded by solute carrier family 2 member 1 (*SLC2A1*) and the major transporter for glucose uptake, was the most conspicuous after TRAF2 knockdown (**Figure 6A**). In addition, we found that TRAF2 siRNA significantly decreased GLUT1 protein expression (**Figure 6B**), which confirmed our proteomic results of the downregulation of GLUT1 by TRAF2 siRNA (data not shown), but this effect was reversed when TRAF2 plasmid was administered (**Figure 6C**). Nevertheless, siRNA to GLUT1 or overexpression of GLUT1 did not affect TRAF2 levels compared to controls in LX-2 cells (**Figure S13A and S13B**). The data suggest that TRAF2 lies upstream of GLUT1. Inhibition of GLUT1 was reported to be able to exhibit an anti-HSC activation and fibrotic activity 40. To further interrogated the results, we used LX-2 cells to study and confirmed that, GLUT1 siRNA blocked the protein levels of HSC activation and fibrosis-correlated markers examined by immunoblotting in comparison to Ctrl siRNA (**Figure S14A**). Besides, cell proliferation rate was notably declined when GLUT1 siRNA was administered, as evidenced by counting the number of LX-2 cells incorporating EdU into their DNA (**Figure S14B**). We also applied a pharmacological strategy to inhibit GLUT1 activity by using small molecule inhibitor BAY-876 to study its antifibrotic effect. Similarly, the levels of molecules associated with HSC activation and fibrosis were prominently reduced in a dose-dependent manner by BAY-876 (**Figure S14C**). Furthermore, silencing GLUT1 expression with siRNA in LX-2 cells prevented the up-regulated expression in α-SMA and COL1A1 protein caused by TRAF2 overexpression (**Figure 6D**). The findings above highlighted that TRAF2 facilitated HSC activation by the upregulation of GLUT1. Increased translocation of GLUTs from intracellular environment to plasma membrane (PM) reportedly could promote glucose transport 41-43. Interestingly, our immunofluorescence experiments also revealed that overexpression of TRAF2 not only enhanced the MFI of total GLUT1 molecules in LX-2 cells, but also facilitated the movement of endogenous GLUT1 to PM (**Figure S15**).

HIF-1α, identified as a regulatory transcription factor, is known to exhibit a direct impact in transcribing numerous genes linked to glycolysis 44. Additionally, GLUT1 has been proven to be a transcriptional target of HIF-1α 45. To further investigate whether GLUT1 expression upregulated by TRAF2 was associated with HIF-1α, we first verified that TRAF2 knockdown or overexpression inhibited or accelerated HIF-1α protein levels in LX-2 cells, respectively (**Figure 6E and 6F**). Given the importance of HIF-1α on the transcriptional expression of GLUT1, we also determined and discovered that silencing TRAF2 expression led to decreased levels of hexokinase 2 (HK2) and the lactate transporter monocarboxylate transporter 4 (MCT4), two reportedly transcriptional targets of HIF-1α (**Figure S16A and S16B**). In addition, we found the declined nuclear translocation of HIF-1α protein after knockdown of TRAF2 compared with the Ctrl siRNA group (**Figure 6G**). A luciferase reporter assay further confirmed that the transcriptional activity of HIF-1α showed a marked increase by co-transfection of TRAF2 (**Figure 6H**). Moreover, an immunoblotting assay demonstrated that overexpression of TRAF2 enhanced boosted GLUT1 protein expression, which was abolished by the siRNA to HIF-1α treatment (**Figure 6I**). Together, these findings revealed that TRAF2 primarily enhanced GLUT1 expression by means of the regulation of HIF-1α.

Finally, to investigate whether HSC-specific ablation of TRAF2 inhibited GLUT1 and HIF-1α expression in animal models, we conducted the double immunofluorescence staining on liver tissue slices from CCl_4_-induced liver fibrosis models in transgenic mice, as shown in the **Figure S17**. Consistent with the *in vitro* data, the levels of GLUT1 and HIF-1α were significantly lower in α-SMA-positive cells of TRAF2^△HSC^ mice than TRAF2^f/f^ mice after CCl_4_ treatment.

### TRAF2 upregulates HIF-1α expression by blocking its degradation mediated by ubiquitin-proteasome pathway

**Figure 7:**
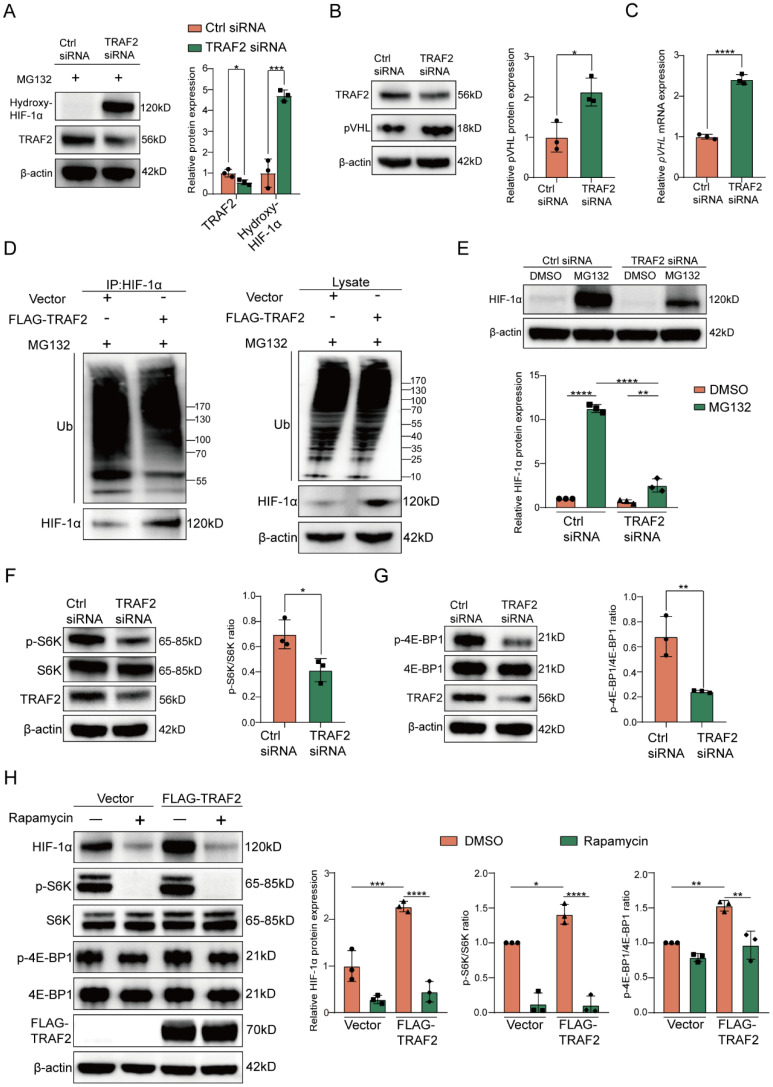
** TRAF2 upregulated HIF-1α levels by obstructing pVHL-dependent proteasomal degradation of HIF-1α and activating mTORC1-mediated increased HIF-1α translation.** (A) Immunoblotting analyses of protein levels of hydroxylated HIF-1α in LX-2 cells transfected with or without TRAF2 siRNA for 48 h, followed by a 6 h-treatment of MG132 (10 µM). (B) Immunoblotting analyses of pVHL protein level in LX-2 cells silencing TRAF2 or not. (C) Determination of the relative mRNA level of *pVHL* in LX-2 cells silencing TRAF2 or not. (D) After a 6 h-treatment of MG132 (10 µM), IP was performed on LX-2 cells overexpressing TRAF2 or not using antibody against HIF-1α and protein A/G agarose. Precipitated proteins and whole-cell lysates were analyzed using indicated antibodies. (E) Immunoblotting analyses of HIF-1α protein level in LX-2 cells. (F) Representative bands of p-S6K and S6K in LX-2 cells silencing TRAF2 or not were determined by immunoblotting. (G) Representative bands of p-4E-BP1 and 4E-BP1 in LX-2 cells silencing TRAF2 or not were measured by immunoblotting. (H) Elevated protein levels of HIF-1α, p-S6K and p-4E-BP1 induced by TRAF2 overexpression were blocked by rapamycin. All data are represented as the means ± SD. *, P<0.05; **, P<0.01; ***, P<0.001; ****, P<0.0001. **Abbreviations**: TRAF2: Tumor necrosis factor receptor-associated factor 2; HIF-1α: Hypoxia-inducible factor-1α; pVHL: von Hippel-Lindau; Ub: Ubiquitin; S6K: p70 ribosomal S6 kinase; 4E-BP1: eukaryotic translation initiation factor 4E (eIF4E)-binding protein 1.

As is known to us, HIF-1α is subjected to prolyl hydroxylase domain (PHD) proteins-induced oxygen-dependent hydroxylation at its proline residues, and the prolyl hydroxylation causes a conformational alteration of HIF-1α, which further allows it to bind to the von Hippel-Lindau (pVHL) protein for degradation through ubiquitin-proteasome pathway 46-49. To investigate whether the effect of TRAF2 on HIF-1α protein levels had relevance to the ubiquitin-proteasome pathway, we first verified the impact of TRAF2 knockdown on hydroxylated HIF-1α in LX-2 cells supplemented with MG132, a proteasome inhibitor. The findings indicated that the presence of MG132 contributed to higher hydroxylated HIF-1α protein levels in LX-2 cells that expressed TRAF2 at a low level in comparison to the control group (**Figure 7A**). We also found that knockdown of TRAF2 could upregulate pVHL protein and mRNA levels (**Figure 7B and 7C**). Furthermore, under MG132 treatment, TRAF2 overexpression decreased the ubiquitination of HIF-1α, but had little influence on ubiquitin levels in whole-cell lysates (**Figure 7D**). Likewise, in the presence of MG132, knockdown of TRAF2 significantly increased ubiquitin proteins in immunoprecipitates pulled down by the HIF-1α antibody (**Figure S18**). In order to delve deeper into the biological regulatory function of TRAF2 in the proteasomal degradation of HIF-1α, LX-2 cells preprocessed with TRAF2 siRNA or Ctrl siRNA for 48 h were exposed to MG132 for 6 h. Interestingly, regardless of whether TRAF2 expression was inhibited in LX-2 cells, treatment with MG132 predisposed to a rise in HIF-1α levels compared to the MG132-untreated cells (**Figure 7E**). Additionally, we observed a much lower HIF-1α level after TRAF2 knockdown with MG132 treatment, as compared to Ctrl siRNA-MG132 treated cells (**Figure 7E**). Totally, these data illustrate that TRAF2 has a regulatory effect on the ubiquitination and proteasomal degradation of HIF-1α, which is ultimately conducive to its stabilization.

### TRAF2 upregulates HIF-1α expression by activating mTORC1 pathway

Based on the KEGG enrichment analysis above, there was a marked inhibition in the activation of PI3K-AKT signaling pathway when TRAF2 siRNA was administered into LX-2 cells in comparison to the Ctrl siRNA group (**Figure 5H**). Previous studies have shown that mTORC1, the downstream molecule of AKT, promotes the translation of metabolic enzymes and metabolism-related transcription factors by phosphorylation of its downstream substrate p70 ribosomal S6 kinase (p70-S6K or S6K) or eukaryotic translation initiation factor 4E (eIF4E)-binding protein 1 (4E-BP1) 50-54. Therefore, we hypothesized that TRAF2 increased HIF-1α synthesis via activating mTORC1 signaling pathway in addition to the decreased proteasomal degradation of HIF-1α. We observed that mTORC1 activity was remarkably downregulated in LX-2 cells silencing TRAF2, as evidenced by a reduction in the levels of phosphorylated S6K (p-S6K) and phosphorylated 4E-BP1 (p-4E-BP1) (**Figure 7F and 7G**). Moreover, we confirmed that the up-regulated levels of p-S6K, p-4EBP1 and HIF-1α induced by TRAF2 overexpression were abrogated when rapamycin (an mTORC1 inhibitor) was added to LX-2 cells (**Figure 7H**). In general, these findings support our hypothesis that mechanistically, TRAF2 can indeed upregulate HIF-1α protein synthesis by promoting the mTORC1 signaling pathway.

## Discussion

**Figure 8:**
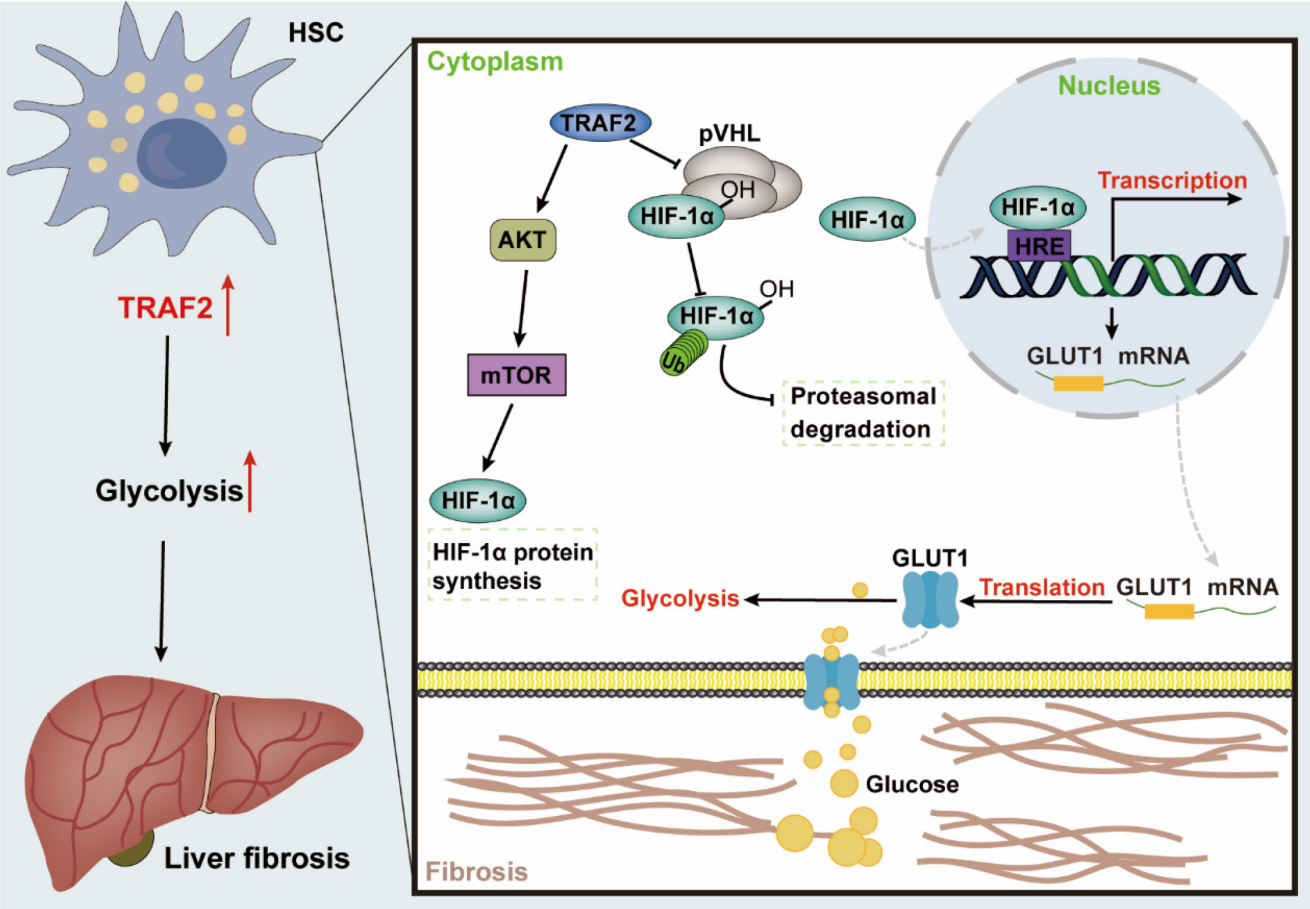
** Graphical abstract explained the mechanism of TRAF2 promoting HSC activation and liver fibrosis development.** TRAF2 promoted HIF-1α translation by mediating mTORC1 signaling and increased HIF-1α protein stability by inhibiting its pVHL-dependent degradation. HIF-1α recognized the HRE of DNA after nuclear translocation, which facilitated the transcriptional expression of several glycolytic proteins, such as glucose transporter GLUT1. Enhanced GLUT1 expression and translocation to the PM facilitated glucose uptake of HSCs, which in turn promoted glycolysis and aggravated the development of liver fibrosis. **Abbreviations**: HSC: Hepatic stellate cell; TRAF2: Tumor necrosis factor receptor-associated factor 2; AKT: Serine/threonine kinase; mTOR: Mammalian target of rapamycin; mTORC1: mTOR Complex 1; HIF-1α: Hypoxia-inducible factor-1α; pVHL: von Hippel-Lindau; HRE: Hypoxia response element; GLUT1: Glucose transporter 1; DNA: Deoxyribonucleic acid; PM: Plasma membrane.

Fibrosis may occur in multiple organs, with increased ECM and myofibroblast-rich scar tissues gradually driving parenchymal dysfunction as its main pathologic manifestation, which poses a serious threat to human health 55. Liver fibrosis represents the common precursor shared by a variety of serious liver diseases and complications 1, 56. Currently, public awareness of the importance of anti-fibrosis therapy is raised, nevertheless, the development of effective anti-fibrogenic agents remains a pressing barrier to clinical 57, 58. Herein, our research revealed that TRAF2 played a crucial role in regulating glycolysis involved in HSC pro-fibrosis reaction, and might represent a therapeutic target for liver fibrosis. Mechanically, TRAF2 sustained the stability of HIF-1α via decreasing its ubiquitination modification-induced proteasomal degradation and activated mTORC1-mediated increased HIF-1α translation, ultimately promoting glycolysis to initiate a profibrogenic response in HSCs (**Figure 8**).

TRAF2, a representative member of the TRAF family, is recognized for its biological role as a scaffold protein in the signal transduction of pro-inflammatory signaling. Studies about the correlation between TRAF2 and liver diseases have been reported almost entirely on hepatitis and liver cancer, thus, we attempted to decipher the function of TRAF2 on the pathogenesis of liver fibrosis. Firstly, unlike healthy liver tissues, high expression level of TRAF2 in clinical and rodent samples of liver fibrosis/cirrhosis was detected. We noted that in fibrotic liver tissues, enhanced fluorescence signal of TRAF2 was primarily localized to hepatocytes and HSCs. In view of the fact that HSCs contribute to the majority of the pool formation of myofibroblast and represent the key cell type driving liver fibrosis, hence, our main interest herein is to investigate the involvement and possible molecular mechanism of TRAF2 in HSC activation during liver fibrosis. By isolating and culturing primary mouse HSCs, we found that TRAF2 expression was increased during the activation of HSCs, but TRAF2 expression was downregulated as the number of activated HSC declined through reversion to an inactivated phenotype induced by Matrigel. Moreover, we revealed that TRAF2 could directly regulate HSC activation and fibrotic phenotype using both loss-of-function and gain-of-function models *in vitro*. Using TRAF2^ΔHSC^ mice* in vivo*, we demonstrated that the ablation of TRAF2 in HSCs protected mice against CCl_4_- and BDL-induced liver fibrosis, further supporting the idea that TRAF2 is a direct regulator during liver fibrogenesis.

In cases of prolonged liver damage, quiescent HSCs transdifferentiate into myofibroblasts and acquire a range of proliferative and pro-fibrotic phenotypes, which is a high energy-demanding process 37. The reprogramming of HSC glycolysis is one of the most crucial metabolism properties of liver fibrosis, with aerobic glycolysis serving as a significant energy source for HSC activation, akin to the Warburg effect present in cancer cells 37. Under this condition, the surplus pyruvate is transferred to the cytosol for conversion to lactate, rather than being transported to the mitochondria for the involvement of the tricarboxylic acid cycle 53. Earlier studies have indicated the relationship between aerobic glycolysis and liver fibrogenesis 40, 59-61. Reportedly, preventing HSC activation and liver fibrosis development could be achieved by inhibiting 6-phosphofructo-2-kinase/fructose-2,6-bisphosphatase-3 (PFKFB3) and by either specifically targeting HK2 in HSCs or systemically eliminating HK2^59^, ^61^. Therefore, interventions targeting glycolysis may help to generate new perspectives for clarifying the molecular mechanism of liver fibrosis and arise a greater possibility for proposing more effective anti-fibrosis strategies. Previous studies have provided some clues that TRAF2 seems to be relevant to glucose metabolism. As firmly established in the literature, intrahepatic TRAF2 was able to exert influence on hepatic glucose metabolism by significantly increasing gluconeogenesis 62. In addition, recruitment of TRAF2 and TRAF6 by mitochondrial antiviral signaling proteins (MAVS) to mitochondria-associated endoplasmic reticulum membrane (MAM) formed the MAVS signalosome forms that drove glycolysis toward HBP and type I interferon expression 63. Our work unveiled a previously unrecognized regulatory mechanism in the context of liver fibrosis that influenced glycolytic activity in HSCs through the function of TRAF2. We found that knockdown of TRAF2 in LX-2 cells was accompanied by the inhibition of glycolytic activity, mainly manifested by the significant diminishment in glucose uptake and corresponding lactate generation of cells and the expression of most glycolysis-related molecules. Notably, TRAF2-mediated abnormal glycolytic reprogramming facilitated HSC activation readily, accelerating the progression of liver fibrosis. Glucose transport is the first rate-limiting step that controls the production of adenosine triphosphate (ATP) in the overall process of energy metabolism. Our results showed that the key glucose transporter GLUT1 expression at mRNA and protein levels was downregulated by TRAF2 siRNA, whereas increased in LX-2 cells overexpressing TRAF2. GLUT1 was reportedly highly overexpressed* in vivo* and *in vitro* models of liver fibrosis and human fibrotic liver tissue samples 37, 40. Importantly, changes in GLUT1 are not only act as a bystander in the evolution of the profibrotic phenotype of HSCs from a resting state, but also contribute to activation. The attenuation of GLUT1 was proven to effectively prohibit HSC proliferation, activation, and fibrotic phenotype, as we demonstrated in this study 40. A study showed that TRAF4, which also belongs to the TRAF family, affected glucose metabolism by enhancing the levels of GLUT1 and HK2, suggesting that TRAF family might exert their regulatory functions in glycolysis via targeting GLUT1 64. In particular, the upregulated expression of markers related with HSC activation and fibrotic phenotype induced by TRAF2 overexpression was blocked by GLUT1 siRNA, indicating that TRAF2 promoted glycolysis mainly through the upregulation of GLUT1, thereby inducing liver fibrogenesis. We also noted that in addition to affecting GLUT1 expression, overexpression of TRAF2 recruited more GLUT1 proteins from the cytoplasm to the PM, indicating the complex and diverse regulation of GLUT1 by TRAF2. This is one of the issues that we will keep sustained attention and delve deeper.

Our study further clarified the in-depth mechanism by which upregulated TRAF2 in HSCs promoted glycolysis based on the fact with many cases reported in the literature that HIF-1α induced glycolysis by binding to the hypoxia-response element (HRE) of the gene promoter to increase transactivation of glycolysis-related genes 45, 65-68. Previous studies have demonstrated that intensified levels of HIF-1α participates in HSC activation by mediating the transcriptional activation of glycolytic genes 69. Selective inhibition of HIF-1α and glycolytic enzymes in HSCs has been shown to effectively inhibit liver fibrosis 70. In our study, elevated HIF-1α expression by TRAF2 overexpression was observed, which would subsequently boost the translocation of HIF-1α into the nucleus and prompt its transcriptional regulatory function. Moreover, TRAF2 protein overexpression was not accompanied by the upregulation of GLUT1 owing to the addition of HIF-1α siRNA, indicating that TRAF2 indeed promoted GLUT1 expression by regulating HIF-1α expression. Our current study is the first report of identifying HIF-1α as an important regulator in TRAF2-mediated HSC activation and liver fibrosis, broadening the content of previous reports on the relationship between HIF-1α and the pathogenesis of liver fibrosis. After identifying the impact of TRAF2 on HIF-1α expression, another significant discovery that emerged from our study was the clarification of this underlying mechanism mediated by TRAF2. The accumulation of HIF-1α protein is regulated at different levels throughout its life cycle within the cells. The oxygen-dependent regulation of the HIF-1α pathway is known to be mediated by pVHL via the ubiquitin-proteasome pathway 71. Several studies have reported other molecules-mediated degradation of HIF-1α in an oxygen- independent way that contributes considerably to influencing HIF-1α protein levels 71. Moreover, heat shock protein (Hsp90) was known to induce some conformational changes of the structure of HIF-1α, thus initiating its transactivation 71. Meanwhile, Hsp90 has also been reported to protect HIF-1α against the pVHL-independent degradation 71. Our current results confirmed that TRAF2 could reduce hydroxylation and ubiquitination levels of HIF-1α, as well as pVHL expression, implying that TRAF2 stabilized HIF-1α by obstructing its pVHL-dependent ubiquitin-proteasomal degradation. Furthermore, mTORC1 also has been reported to influence HIF-1α synthesis at the translational level by its downstream effector proteins, S6K and 4E-BP1^50^-^52^. To assess the relevance of increased HIF-1α levels induced by TRAF2 overexpression to mTORC1 activation, the mTORC1 inhibitor rapamycin was administered into TRAF2-overexpressed LX-2 cells. As a result, we observed that TRAF2 overexpression led to increased p-S6K and p-4E-BP1 compared to controls, whereas this effect was reversed when rapamycin was added. Simultaneously, rapamycin significantly diminished HIF-1α levels in LX-2 cells exposed to TRAF2 plasmid. Therefore, it is reasonable to infer that the higher abundance of HIF-1α induced by TRAF2, is likely due, in part, to the activation of the mTORC1 pathway.

There are, in addition, limitations to the study that should be noted. Firstly, our study found that the ablation of TRAF2 in hepatocytes mitigated collagen deposition under the treatment of CCl_4_ or BDL. Several studies reported that TRAF2 in hepatocytes promoted hepatic steatosis, insulin resistance, and inflammation 62, 72, 73. Since steatosis and inflammation can increase the risk of HSC activation and liver fibrosis, TRAF2 in hepatocytes may also be linked to the occurrence or progression of liver fibrosis. Furthermore, it has been recognized that hepatocytes can interact directly with HSCs or depend on the crosstalk between cells indirectly to influence HSC activation 32, 74, 75. For example, hepatocyte-derived CXCL10 has been proven to play a crucial role to trigger HSC activation via the crosstalk between hepatocytes and HSCs 27, 32. Hence, high expression of TRAF2 in hepatocytes promoting HSC activation is likely related to the "information exchange" between different cells. However, it is not clear which "information" produced by hepatocytes participates in the TRAF2-mediated effect on liver fibrosis, which is the direction of our future research. Secondly, further mechanism exploration about TRAF2 blocking HIF-1α degradation and promoting HIF-1α translation is also clearly warranted. Thirdly, our data focus on the downstream targets of TRAF2, whereas how TRAF2 is activated during hepatic fibrogenesis still needs further investigation.

## Conclusion

In summary, our work first unmasked the association of TRAF2 and glycolysis in liver fibrosis, highlighting the importance of TRAF2 in regulating the glycolytic flux of HSCs during the progression of liver fibrosis. Mechanistically, we confirmed that TRAF2 regulated glycolysis in liver fibrosis via HIF-1α/GLUT1 axis, thereby inducing liver fibrosis. The in-depth study revealed that TRAF2 in HSCs promoted HIF-1α stability by inhibiting its degradation mediated by pVHL and induced the protein synthesis of HIF-1α through the activation of mTORC1 pathway. This study broadens the effect of TRAF2 on liver diseases and provides a new perspective into the induction of HSC inactivation.

## Supplementary Material

Supplementary figures and tables.
