# Supplementary material for: TRAF2 Promotes Liver Fibrosis via Regulation of the HIF-1α/GLUT1-Mediated Glycolysis in Hepatic Stellate Cells
Source: Int J Biol Sci. 2025 Sep 3;21(13):5645–65. doi: 10.7150/ijbs.99682 (PMC12509682; doi:10.7150/ijbs.99682)
Supplement: Supplementary file 1 — Supplementary figures and tables. [file ijbsv21p5645s1.pdf]

Supporting Information

TRAF2 Promotes Liver Fibrosis Via Regulation Of The HIF-1α/GLUT1-Mediated Glycolysis In Hepatic Stellate Cells

Yina Zhang[#,1,2], Siduo Xu[#,1,2], Jiajia Shao[#,1,2], Yining Lu[1,2], Lingzhu Zhao[1,2], Xue Liang[1,2], Jiping Yao[1,2], Minwei Li[1,2], Yanning Liu*[,1,2] and Min Zheng*[,1,2]

[1] State Key Laboratory for Diagnosis and Treatment of Infectious Diseases, National Clinical Research Center for Infectious Diseases, China-Singapore Belt and Road Joint Laboratory on Infection Research and Drug Development, National Medical Center for Infectious Diseases, Collaborative Innovation Center for Diagnosis and Treatment of Infectious Diseases, The First Affiliated Hospital, College of Medicine, Zhejiang University, Hangzhou, 310003, China.

[2] Yuhang Institute of Medical Science Innovation and Transformation, Hangzhou, 310000, China.

# Yina Zhang, Siduo Xu and Jiajia Shao contributed equally to this work.

* Correspondence:

Yanning Liu   liuyanning@zju.edu.cn

Min Zheng   minzheng@zju.edu.cn

Supplementary Figures

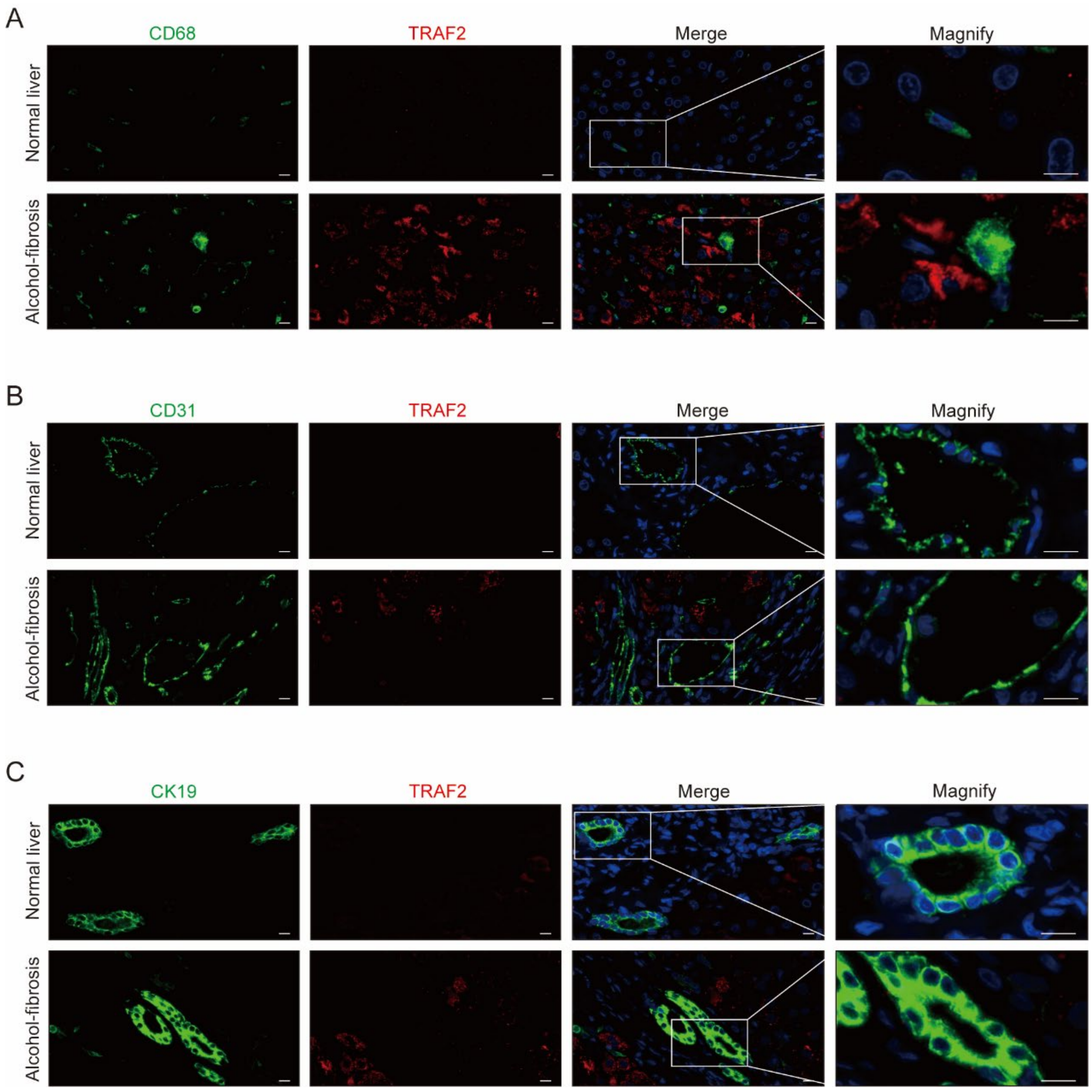

Figure S1. TRAF2 expression in various liver cell types in humans. (A) TRAF2 expression in Kupffer cells. Representative double immunofluorescence images of CD68 (green) and TRAF2 (red) in normal and alcohol-induced fibrotic liver tissues. (B) TRAF2 expression in endothelial cells. Representative double immunofluorescence images of CD31 (green) and TRAF2 (red) in normal and alcohol-induced fibrotic liver tissues. (C) TRAF2 expression in bile duct cells. Representative double immunofluorescence images of CK19 (green) and TRAF2 (red) in normal and alcohol-induced fibrotic liver tissues. Scale bars = 10 μm. **Abbreviations**: CD68: cluster of differentiation 68; TRAF2: Tumor necrosis factor receptor-associated factor 2; CD31: cluster of differentiation 31; CK19: cytokeratin 19.

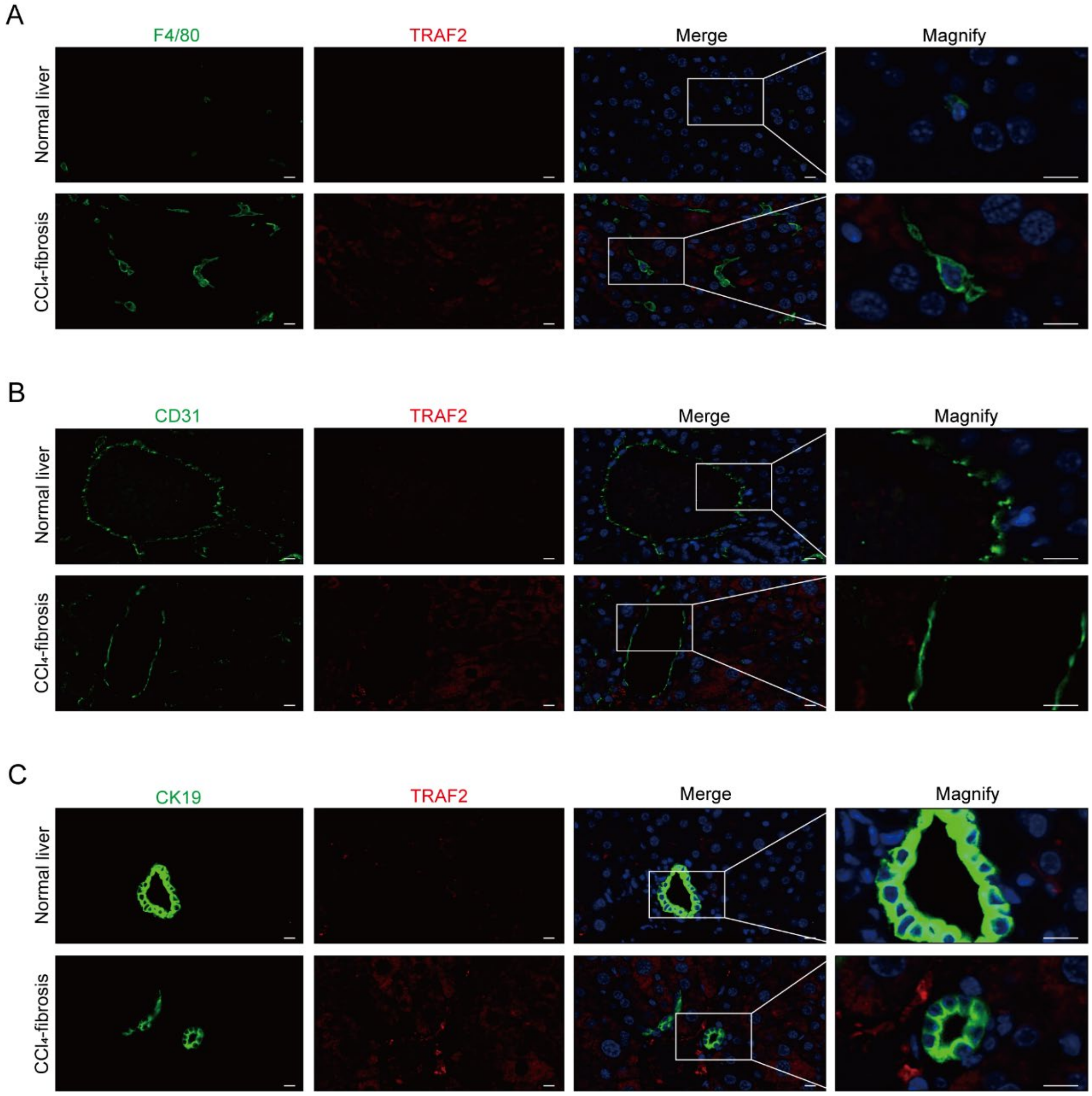

Figure S2. TRAF2 expression in various liver cell types in mice. (A) TRAF2 expression in Kupffer cells. Representative double immunofluorescence images of F4/80 (green) and TRAF2 (red) in normal and CCl_4-induced fibrotic mouse liver tissues. (B) TRAF2 expression in endothelial cells. Representative double immunofluorescence images of CD31 (green) and TRAF2 (red) in normal and CCl_4-induced fibrotic mouse liver tissues. (C) TRAF2 expression in bile duct cells. Representative double immunofluorescence images of CK19 (green) and TRAF2 (red) in normal and CCl_4-induced fibrotic mouse liver tissues. Scale bars = 10 μm. **Abbreviations**: TRAF2: Tumor necrosis factor receptor-associated factor 2; CD31: cluster of differentiation 31; CK19: cytokeratin 19; CCl_4: Carbon tetrachloride.

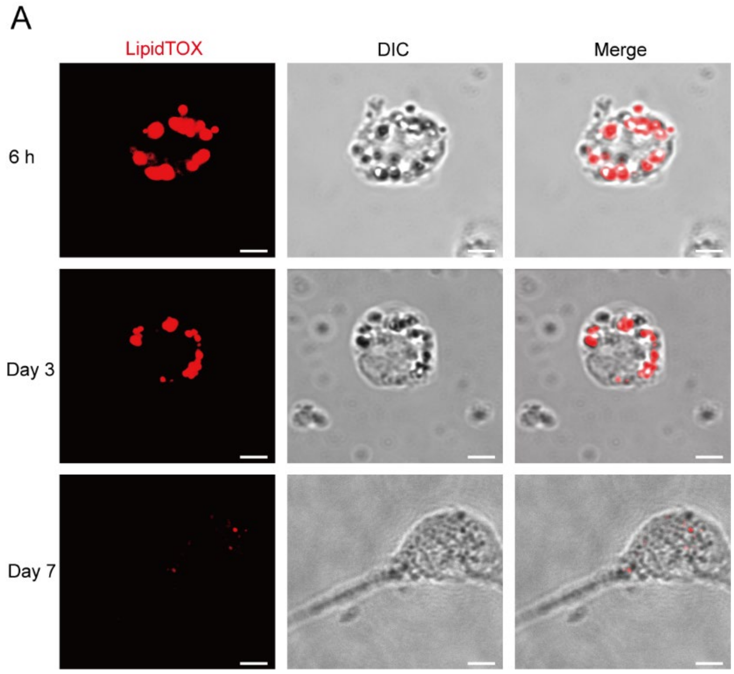

Figure S3. Neutral lipid staining of primary mouse HSCs. (A) Primary HSCs isolated from normal mice were cultured on plastic cell culture dishes for 6 h, 3, or 7 days. Cells were formaldehyde-fixed and labeled with LipidTOX™ neutral lipid stain (red) according to the manual. Scale bars = 3 µm. **Abbreviations**: DIC: Differential interference contrast; HSC: Hepatic stellate cell.

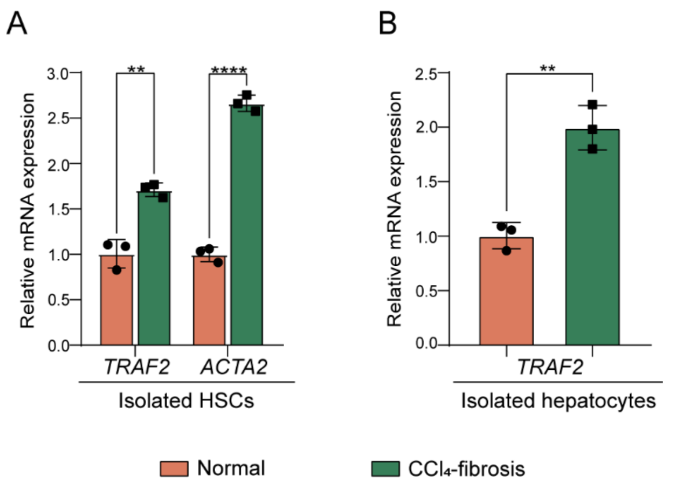

Figure S4. Determination of the relative mRNA expression of *TRAF2* in primary HSCs and hepatocytes isolated from normal and CCl_4-induced fibrotic mouse liver. (A) *TRAF2* and *ACTA2* mRNA expression was elevated in primary HSCs isolated from CCl_4-induced fibrotic mouse liver compared to the normal control. (B) *TRAF2* mRNA expression was elevated in hepatocytes isolated from CCl_4-induced fibrotic mouse liver compared to the normal control. All data are represented as the means ± SD. **, $P < 0.01$; ****, $P < 0.0001$. **Abbreviations**: TRAF2: Tumor necrosis factor receptor-associated factor 2; HSC: Hepatic stellate cell; CCl_4: Carbon tetrachloride.

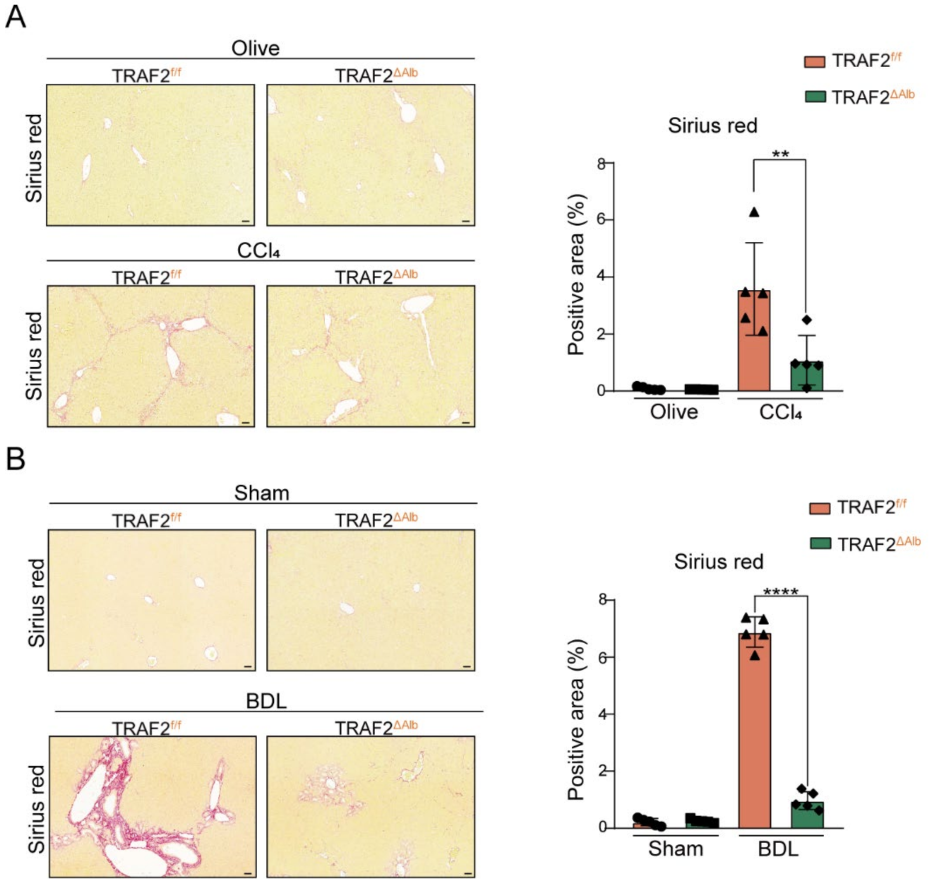

Figure S5. Hepatocytes-specific ablation of TRAF2 alleviated CCl_4- and BDL-induced liver fibrosis in mice. (A) Sirius red staining for ECM deposition was performed on the liver tissues of TRAF2$^{f/f}$ and TRAF2$^{\triangle Alb}$ mice receiving olive or CCl_4 and the corresponding quantification was measured by Image Pro Plus software. Scale bars = 50 μm. (B) Sirius red staining for ECM deposition was performed on the liver tissues of TRAF2$^{f/f}$ and TRAF2$^{\triangle Alb}$ mice receiving sham operation or BDL and the corresponding quantification was measured by Image Pro Plus software. Scale bars = 50 μm. All data are represented as the means ± SD. **, $P < 0.01$; ****, $P < 0.0001$. **Abbreviations**: TRAF2: Tumor necrosis factor receptor-associated factor 2; CCl_4: Carbon tetrachloride; BDL: Bile duct ligation.

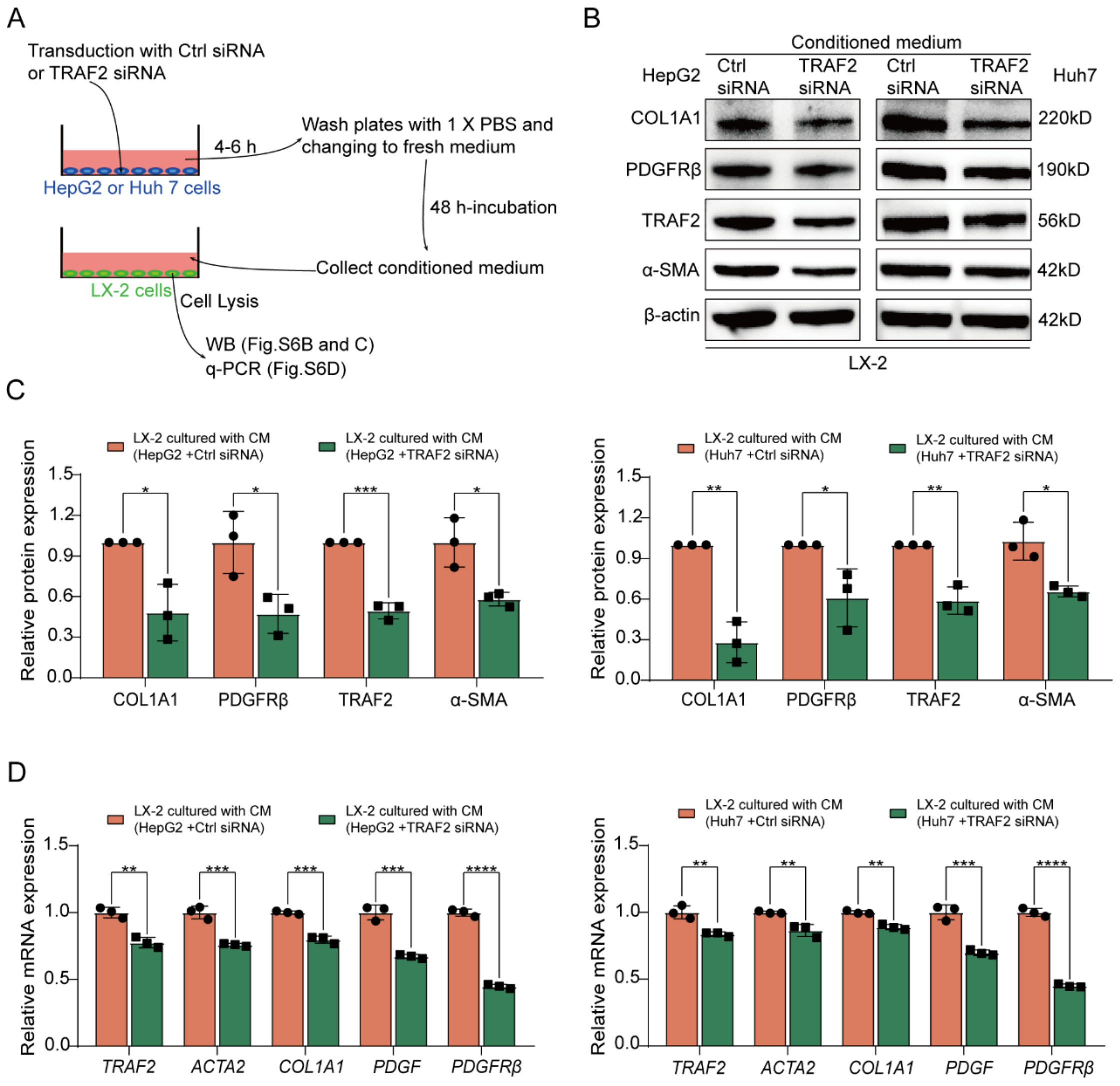

Figure S6. Hepatocyte TRAF2 promotes activation of HSCs *in vitro* via cell-to-cell communication. (A) Schematic illustration of the medium transfer experiment. (B) & (C) After culture with CM derived from TRAF2-knockdown hepatocytes for 24 h, LX2 cells were collected for immunoblotting to assess the protein expression of TRAF2 and fibrosis markers. (D) After culture with CM derived from TRAF2-knockdown hepatocytes for 24 h, LX2 cells were collected for qRT-PCR validation to assess the mRNA expression of *TRAF2* and fibrosis markers. All data are represented as the means ± SD. *, $P < 0.05$; **, $P < 0.01$; ***, $P < 0.001$; ****, $P < 0.0001$. **Abbreviations**: TRAF2: Tumor necrosis factor receptor-associated factor 2; PBS: Phosphate buffer saline; WB: Western blotting; qRT-PCR: Quantitative reverse-transcription polymerase chain reaction; CM: Conditioned medium; α-SMA: α-smooth muscle actin; COL1A1: Collagen type 1; PDGFRβ: Platelet-derived growth factor receptor β; PDGF: Platelet-derived growth factor; HSC: Hepatic stellate cell.

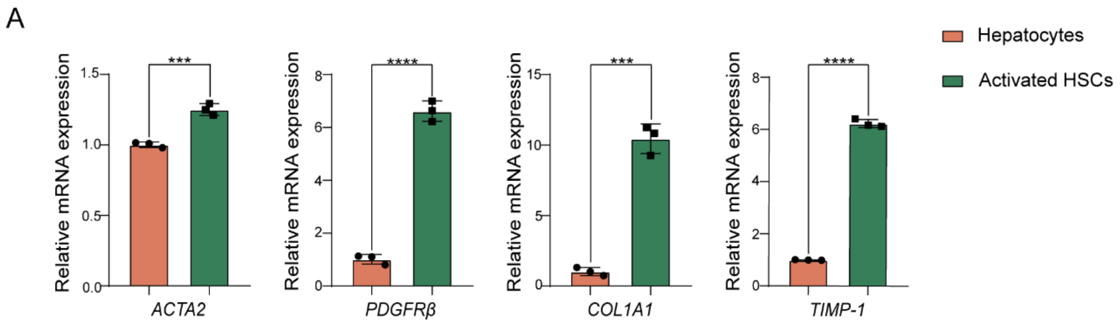

Figure S7. Activated HSCs expressed higher levels of fibrosis-related genes compared to hepatocytes. (A) *ACTA2*, *PDGFRβ*, *COL1A1* and *TIMP-1* mRNA expression was dramatically elevated in primary HSCs isolated from CCl_4-induced fibrotic mouse liver compared to hepatocytes. All data are represented as the means ± SD. ***, $P < 0.001$; ****, $P < 0.0001$. **Abbreviations**: HSC: Hepatic stellate cell; PDGFRβ: Platelet-derived growth factor receptor β; COL1A1: Collagen type 1; TIMP-1: Tissue inhibitor of metalloprotease-1.

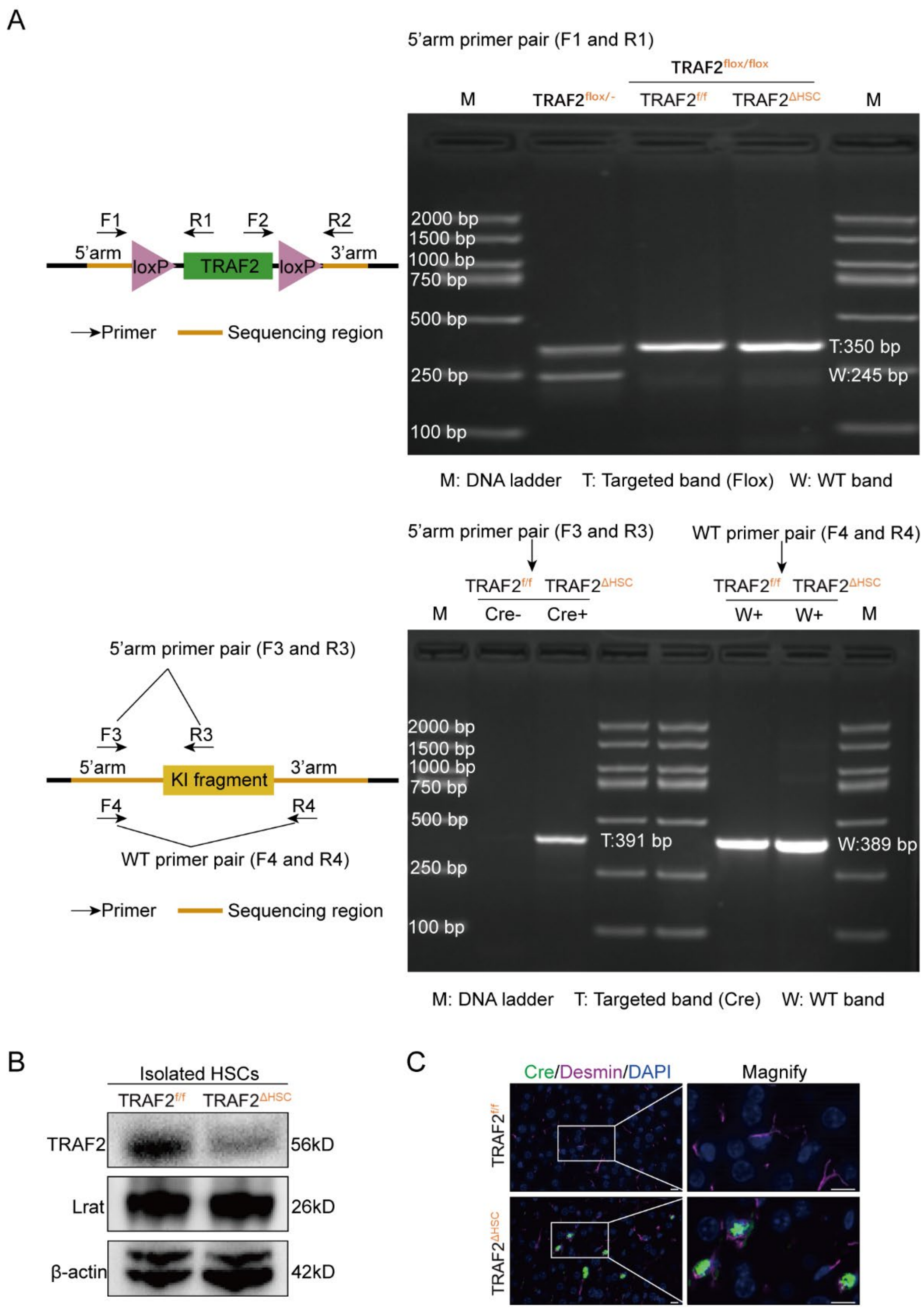

Figure S8. Genotype identification of transgenic mice and verification of the effect of HSC-specific TRAF2 gene knockout. (A) Genotype identification of TRAF2$^{f/f}$ and TRAF2$^{\Delta HSC}$ mice. (B) Immunoblotting confirmation of TRAF2 and Lrat expression in primary HSCs isolated from TRAF2$^{f/f}$ and TRAF2$^{\Delta HSC}$ mice. (C) Double immunofluorescence images of Cre (green) and Desmin (purple) in HSCs isolated from TRAF2$^{f/f}$ and TRAF2$^{\Delta HSC}$ mice. **Abbreviations**: TRAF2: Tumor necrosis factor receptor-associated factor 2; HSC: Hepatic

stellate cell; DNA: Deoxyribonucleic acid; WT: Wild type; Cre: Cyclization recombinant enzyme; Lrat: Lecithin retinol acyl transferase; DAPI: 4,6-diamidino-2-phenylindole.

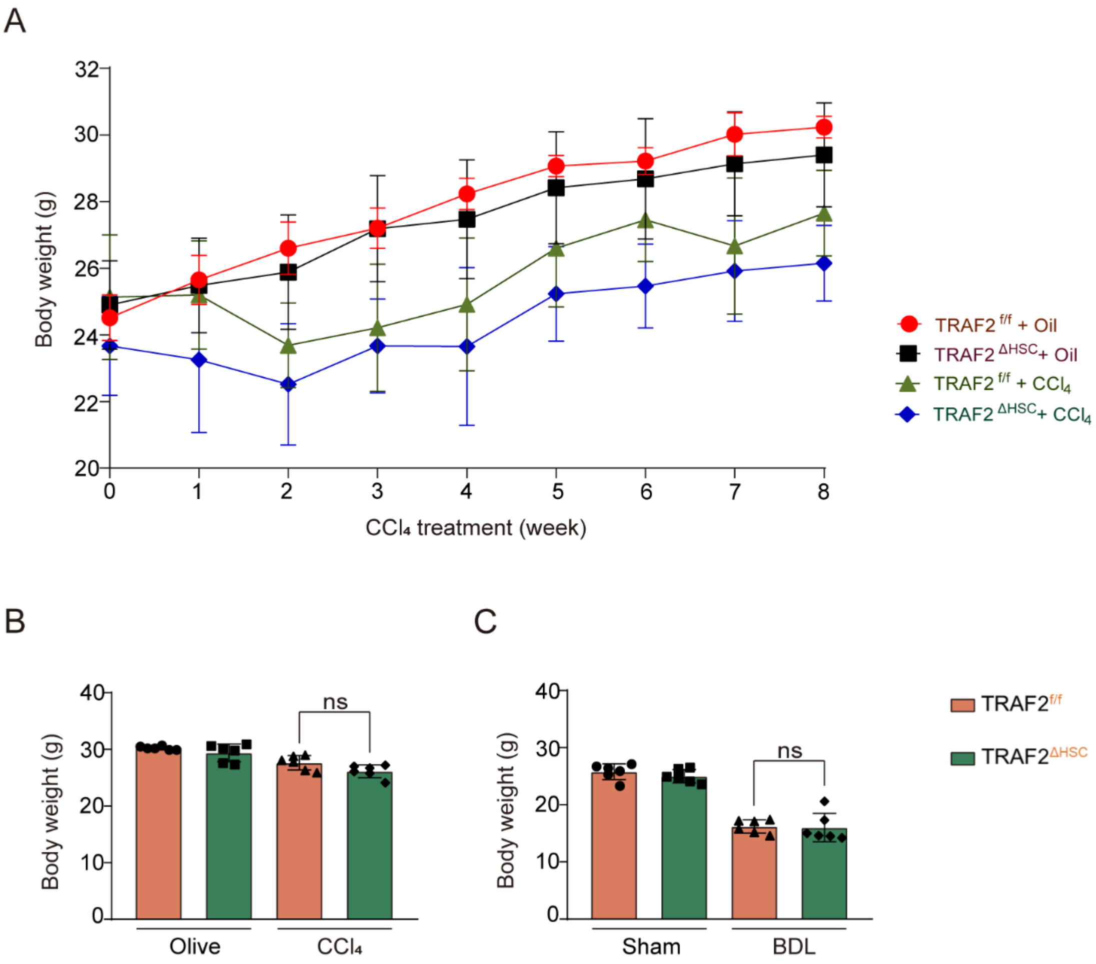

Figure S9. Body weight change for each group during the CCl_4 or BDL treatment period. (A) Body weight change for each group during the 8-week CCl_4 treatment period. (B) Three days after the final CCl_4 injection, the body weight for each group was recorded before the mice were sacrificed. (C) On day 15 after Sham or BDL surgery, all mice were weighed before sacrifice. All data are represented as the means ± SD. ns, not significant. **Abbreviations**: CCl_4: Carbon tetrachloride; BDL: Bile duct ligation; TRAF2: Tumor necrosis factor receptor-associated factor 2; HSC: Hepatic stellate cell.

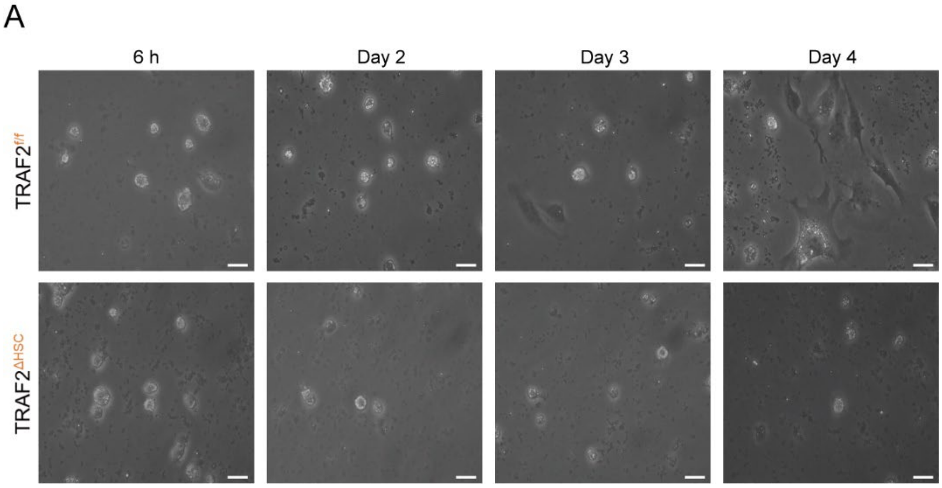

Figure S10. Phase-contrast micrographs of primary mouse HSCs after 6 h, 2, 3, 4 days in culture. (A) Morphological changes of primary HSCs isolated from TRAF2$^{\Delta HSC}$ and TRAF2$^{f/f}$ transgenic mice and cultured on dishes for 6h, 2, 3, 4 days. Scale bars = 20 μm. **Abbreviations**: TRAF2: Tumor necrosis factor receptor-associated factor 2; HSC: Hepatic stellate cell.

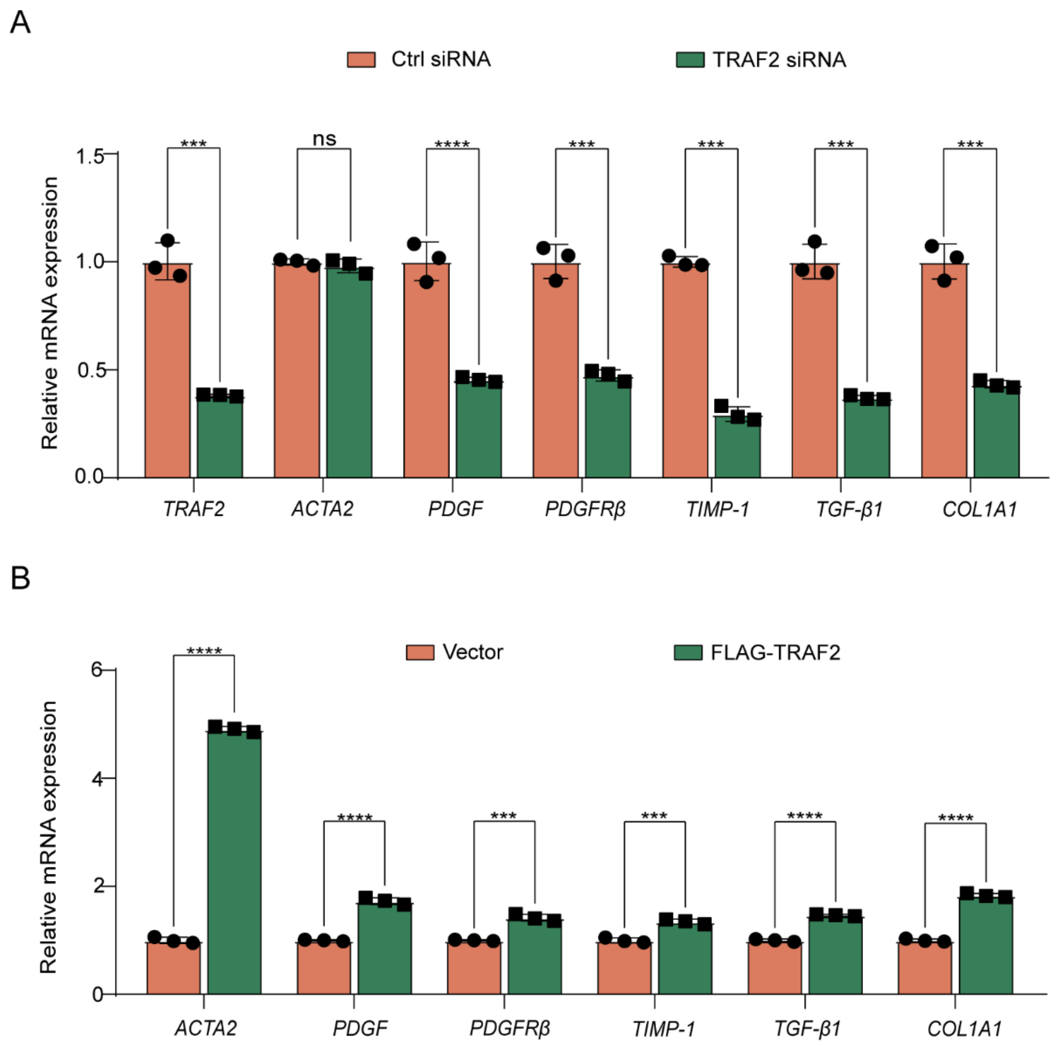

Figure S11. Knockdown of TRAF2 inhibited the mRNA levels of HSC activation and profibrogenic markers. (A) Determination of the mRNA expression of indicated HSC activation and fibrotic markers in LX-2 cells treated with TRAF2 siRNA or Ctrl siRNA. (B) Determination of the mRNA expression of indicated HSC activation and fibrotic markers in LX-2 cells treated with or without TRAF2 plasmid. All data are represented as the means ± SD. ns, not significant; ***, $P < 0.001$; ****, $P < 0.0001$. **Abbreviations**: TRAF2: Tumor necrosis factor receptor-associated factor 2; PDGF: Platelet-derived growth factor; PDGFRβ: Platelet-derived growth factor receptor β; TIMP-1: Tissue inhibitor of metalloprotease-1; TGF-β1: Transforming growth factor β1; COL1A1: Collagen type 1; HSC: Hepatic stellate cell.

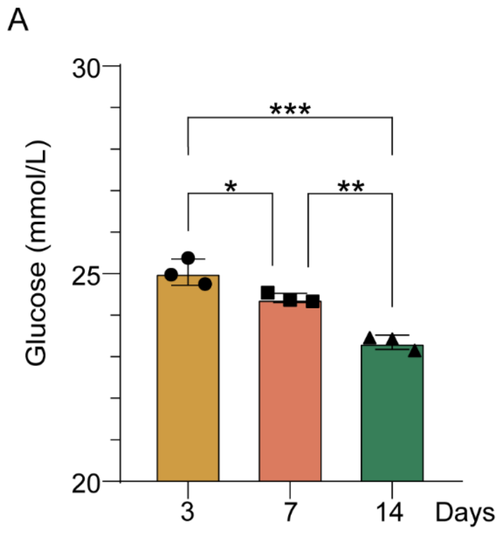

Figure S12. The cell supernatants collected were processed to assess glucose concentration. (A) Primary HSCs were isolated from normal mice and cultured *in vitro* for 3, 7, or 14 days. The glucose levels in the harvested cell supernatant were determined by utilizing a glucose assay kit. All data are represented as the means ± SD. *, $P < 0.05$; **, $P < 0.01$; ***, $P < 0.001$.

Abbreviations: HSC: Hepatic stellate cell.

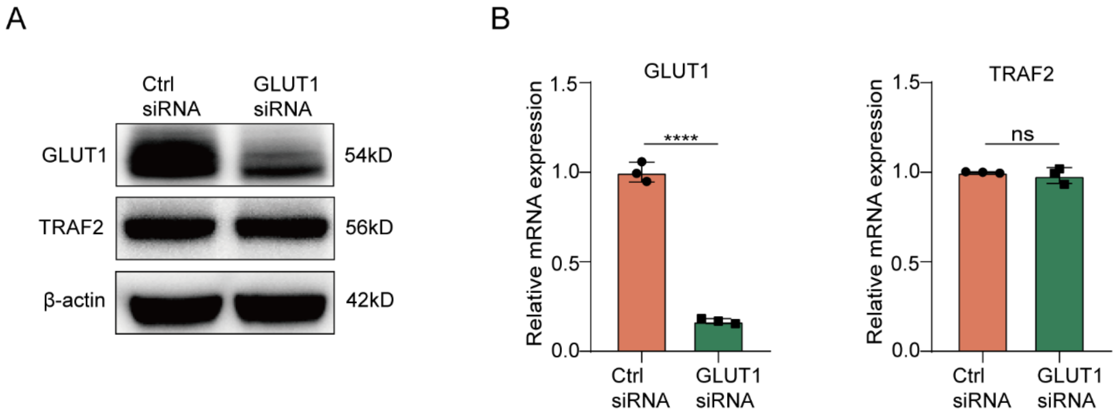

Figure S13. Knockdown of GLUT1 in LX-2 cells did not affect TRAF2 protein and mRNA expression. (A) GLUT1 siRNA did not affect TRAF2 protein expression in LX-2 cells compared to Ctrl siRNA. (B) GLUT1 siRNA did not affect *TRAF2* mRNA expression in LX-2 cells compared to Ctrl siRNA. All data are represented as the means ± SD. ns, not significant; ****, $P < 0.0001$. **Abbreviations**: GLUT1: Glucose transporter 1; TRAF2: Tumor necrosis factor receptor-associated factor 2.

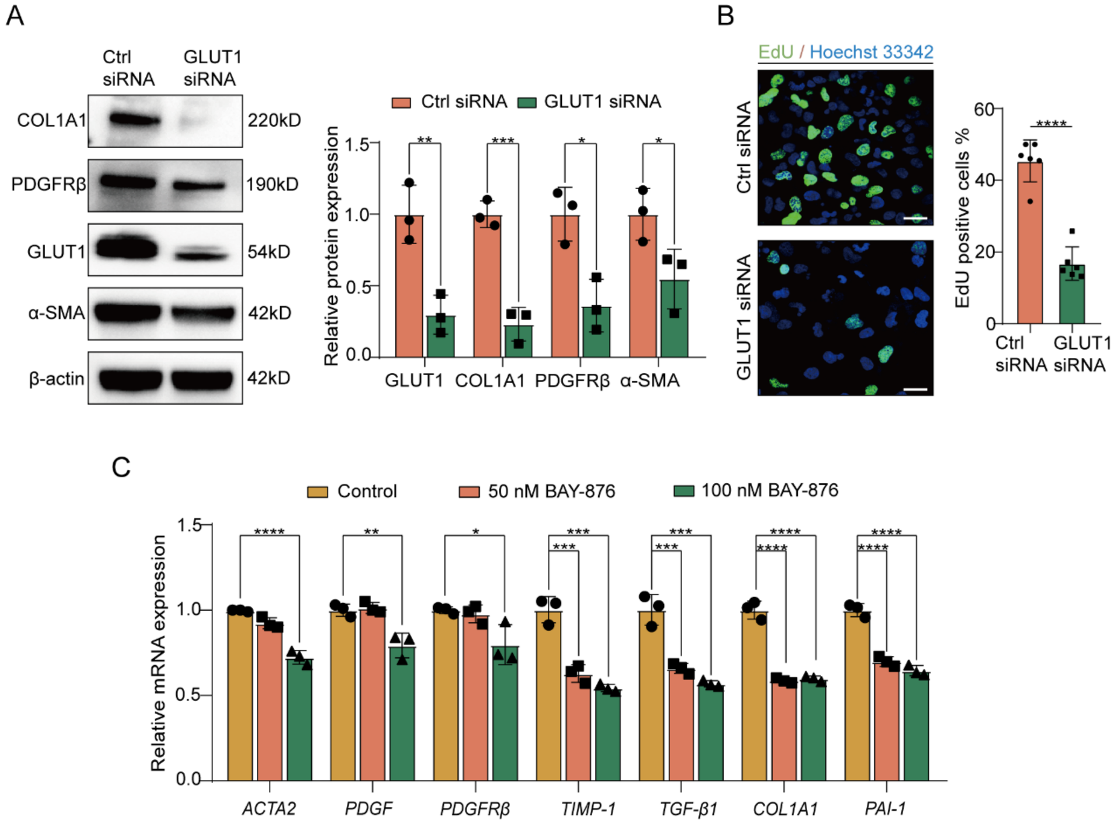

Figure S14. GLUT1 inhibition reduced HSC proliferation, activation and profibrogenic phenotype. (A) Immunoblotting analyses of protein levels of GLUT1, α-SMA, PDGFRβ and COL1A1 in LX-2 cells treated with GLUT1 siRNA or Ctrl siRNA for 48 h. (B) Cell proliferation was detected via EdU staining. Scale bars = 25 μm. EdU% was calculated as EdU-positive (green) cells/total DAPI-positive (blue) cells. (C) Determination of the relative mRNA expression of fibrosis-related markers in LX-2 cells treated with different concentrations of BAY-876. All data are represented as the means ± SD. *, $P < 0.05$; **, $P < 0.01$; ***, $P < 0.001$; ****, $P < 0.0001$. **Abbreviations**: GLUT1: Glucose transporter 1; COL1A1: Collagen type 1; PDGFRβ: Platelet-derived growth factor receptor β; α-SMA: α-smooth muscle actin; EdU: 5-ethynyl-2'-deoxyuridine; PDGF: Platelet-derived growth factor; TIMP-1: Tissue inhibitor of metalloprotease-1; PAI-1: Plasminogen activator inhibitor-1.

A

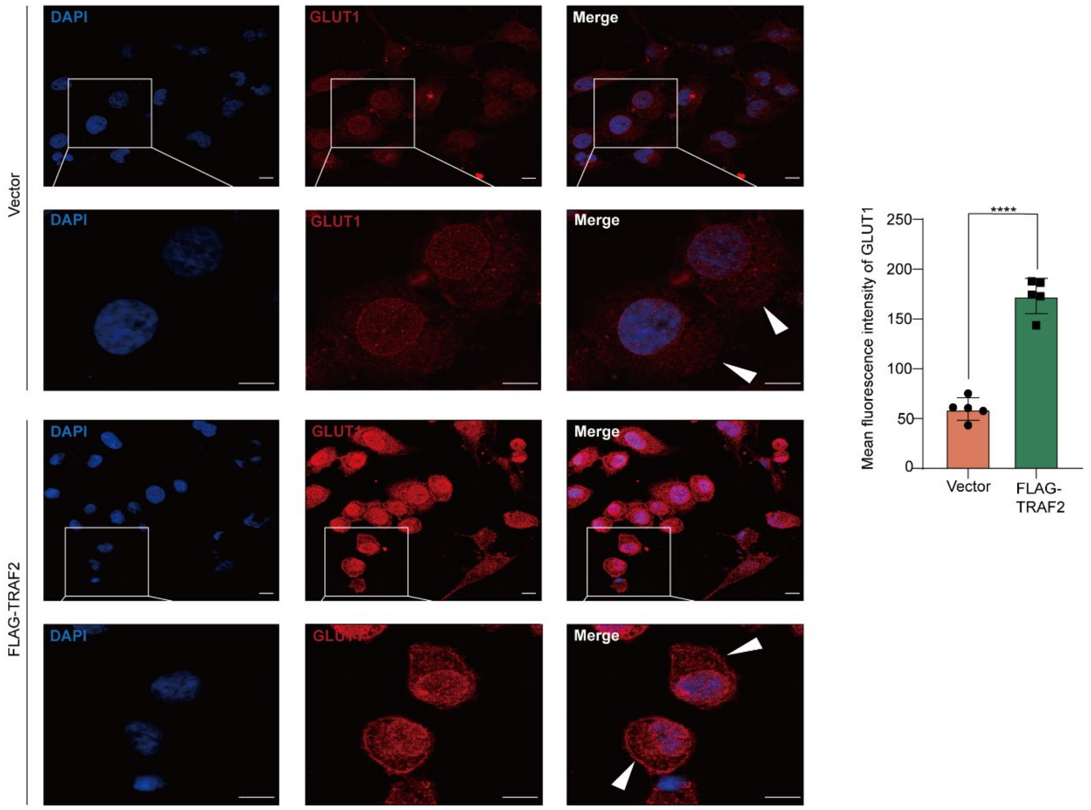

Figure S15. TRAF2 significantly promoted GLUT1 expression and affected GLUT1 localization in HSCs. (A) Overexpression of TRAF2 not only enhanced the mean fluorescence intensity of total GLUT1 in LX-2 cells, but also promoted the translocation of endogenous GLUT1 to the plasma membrane in LX-2 cells. Data are represented as the means ± SD. ****, $P < 0.0001$. **Abbreviations**: DAPI: 4,6-diamidino-2-phenylindole; GLUT1: Glucose transporter 1.

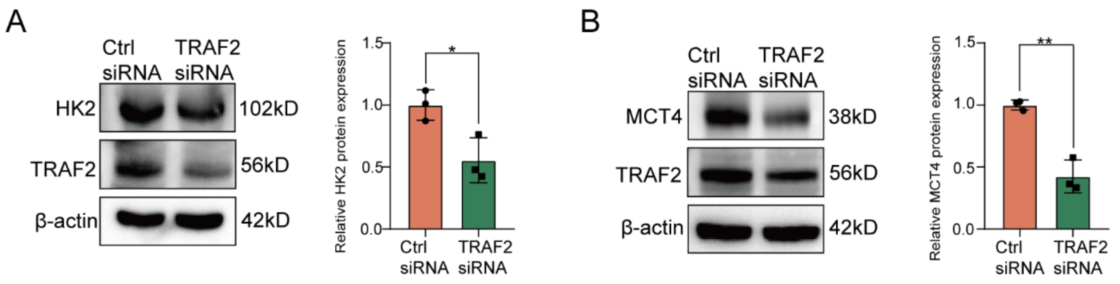

Figure S16. The protein levels of HK2 and MCT4 expression in LX-2 cells treated with or without TRAF2 siRNA for 48 h were tested using immunoblotting. (A) Immunoblotting confirmation of the protein levels of HK2. (B) Immunoblotting confirmation of the protein levels of MCT4. All data are represented as the means ± SD. ns, not significant; *, $P < 0.05$; **, $P < 0.01$. **Abbreviations**: TRAF2: Tumor necrosis factor receptor-associated factor 2; HK2: Hexokinase 2; MCT4: Monocarboxylate transporter 4.

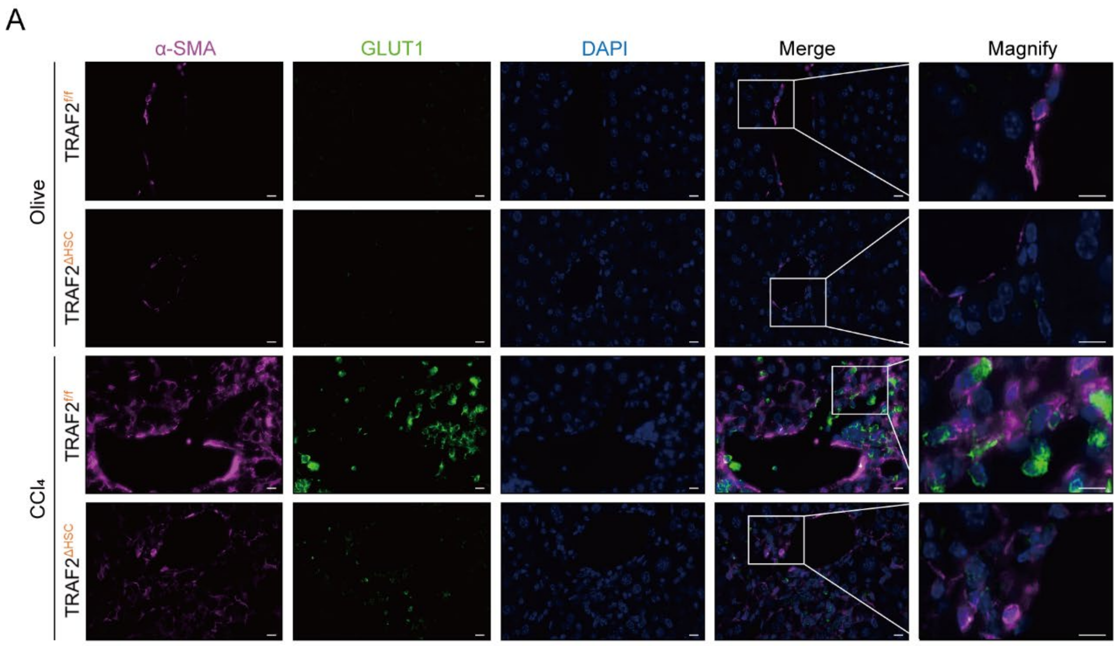

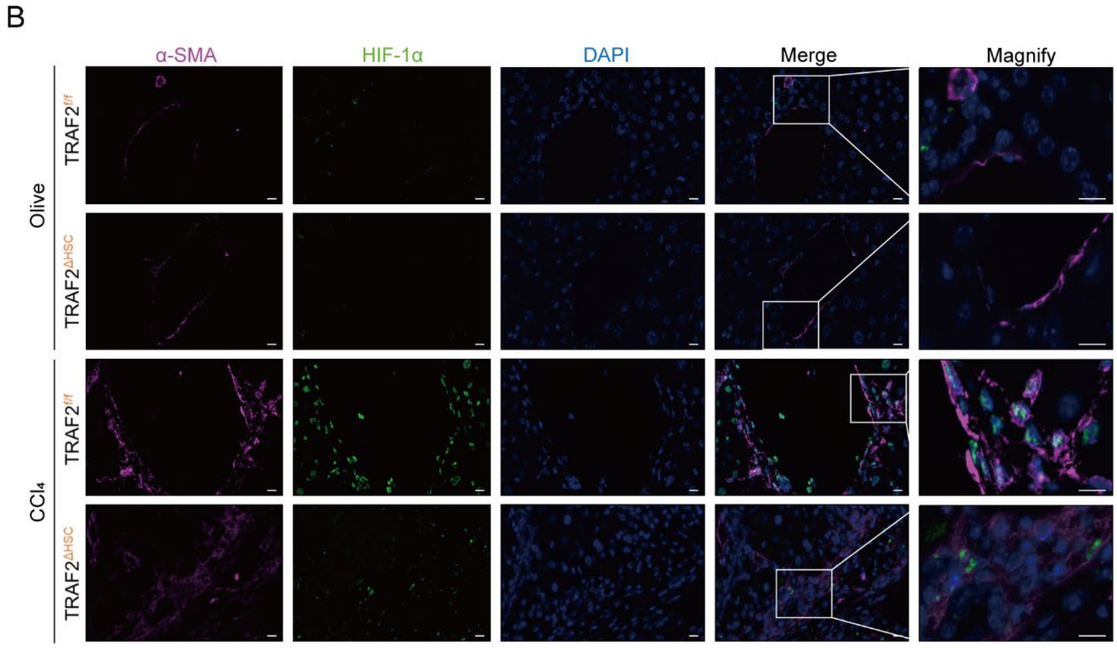

Figure S17. HSC-specific ablation of TRAF2 reduced the levels of GLUT1 and HIF-1α *in vivo* model of liver fibrosis. (A) GLUT1 expression in HSCs. Representative double immunofluorescence images of α-SMA (purple) and GLUT1 (green) in liver samples from CCl_4-induced liver fibrosis models in transgenic mice. (B) HIF-1α expression in HSCs. Representative double immunofluorescence images of α-SMA (purple) and HIF-1α (green) in liver samples from CCl_4-induced liver fibrosis models in transgenic mice. Scale bars = 10 μm. **Abbreviations**: TRAF2: Tumor necrosis factor receptor-associated factor 2; CCl_4: Carbon

tetrachloride; α-SMA: α-smooth muscle actin; GLUT1: Glucose transporter 1; DAPI: 4,6-diamidino-2-phenylindole; HIF-1α: Hypoxia-inducible factor-1α; HSC: Hepatic stellate cell.

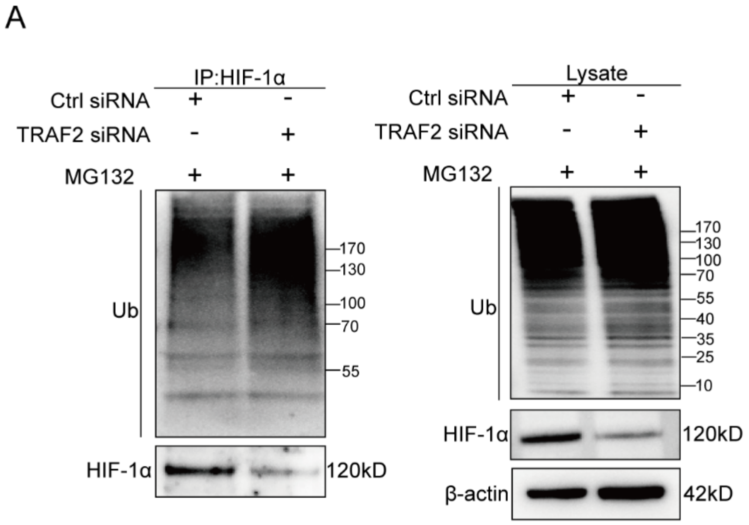

Figure S18. Knockdown of TRAF2 increased the ubiquitination of HIF-1α proteins in presence of MG132. (A) LX-2 cells were transfected with Ctrl siRNA or TRAF2 siRNA for 48 h, followed by a 6 h-treatment of MG132 (10 μM), then, cell lysates of LX-2 cells were immunoprecipitated with HIF-1α antibody and protein A/G agarose. Precipitated proteins and whole-cell lysates were analyzed using indicated antibodies. **Abbreviations**: HIF-1α: Hypoxia-inducible factor-1α; TRAF2: Tumor necrosis factor receptor-associated factor 2; Ub: Ubiquitin.

Supplementary Tables

Table S1. Antibodies information.

Antibody	Application	Source
TRAF2	WB, IHC	Santa cruz (sc-136999; USA)
	IF	Proteintech Group (67315-1-Ig; China)
	IF	Proteintech Group (26846-1-AP; China)
	IF	ABclonal (A0962; China)
α-SMA	WB, IF	CST (#19245; USA)
	WB, IF	Huabio (ET1607-53; China)
	IF, IHC	Servicebio (GB13044; China)
	IF	Affinity Biosciences (AF1032; China)
COL1A1	WB	Huabio (ET1609-68; China)
	IHC	Abcam (ab270993; USA)
PDGFRβ	WB	CST (#28E1; USA)
HNF4α	IF	Boster (PB9215; China)
	IF	ABclonal (A20865; China)
CD68	IF	Servicebio (GB113150; China)
CD31	IF	Abcam (ab182981; USA)
CK19	IF	Abcam (ab52625; USA)
F4/80	IF	CST (#70076; USA)
Lrat	WB	Santa cruz (sc-101391; USA)
Cre	IF	CST (#15036; USA)
Desmin	IF	Proteintech Group (16520-1-AP; China)
GLUT1	IF, WB	Abcam (ab115730; USA)
	IF	Proteintech Group (21829-1-AP; China)
HIF-1α	WB	CST (#36169; USA)
	IP	Proteintech Group (20960-1-AP; China)
	IF	Huabio (HA721997; China)
HK2	WB	Proteintech Group (22029-1-AP; China)
MCT4	WB	Proteintech Group (22787-1-AP; China)
FLAG	WB	CST (#8146; USA)
Hydroxy-HIF-1α	WB	CST (#3434; USA)
pVHL	WB	Proteintech Group (24756-1-AP; China)
p-S6K	WB	Proteintech Group (28735-1-AP; China)
S6K	WB	Proteintech Group (14485-1-AP; China)
p-4E-BP1	WB	Huabio (RT1004; China)
4E-BP1	WB	Huabio (ET1701-83; China)
Histone	WB	Proteintech Group (17168-1-AP; China)
β-actin	WB	Proteintech Group (66009-1-Ig; China)
	WB	ABclonal (AC026; China)
GAPDH	WB	CST (#5174; USA)
Goat anti-mouse IgG-HRP	WB	Huabio (HA1006; China)

Table S1. Antibodies information (continued).

Antibody	Application	Source
Goat anti-rabbit IgG-HRP	WB	Huabio (HA1001; China)
Anti-rabbit IgG-HRP for IP nano-secondary antibody	WB	Huabio (NBI01H; China)
Anti-mouse IgG-HRP for IP nano-secondary antibody	WB	Huabio (NBI02H; China)
Alexa Fluor™ 555 Donkey anti-Rabbit IgG (H+L)	IF	Invitrogen (A31572; USA)
Alexa Fluor™ 488 Donkey anti-Mouse IgG (H+L)	IF	Invitrogen (A21202; USA)

Table S2. Primers for qRT-PCR.

Gene	Sequence (5'-3')	
TRAF2 (hum)	Forward	5'-GCTCATGCTGACCGAATGTC-3'
	Reverse	5'-GCCGTCACAAGTTAAGGGGAA-3'
ACTA2 (hum)	Forward	5'-GTGTTGCCCCTGAAGAGCAT-3'
	Reverse	5'-GCTGGGACATTGAAAGTCTCA-3'
PDGF (hum)	Forward	5'-CTCGATCCGCTCCTTTGATGA-3'
	Reverse	5'-CGTTGGTGCGGTCTATGAG-3'
PDGFRβ (hum)	Forward	5'-GCCCTTATGTCGGAGCTGAAGA-3'
	Reverse	5'-GTTGCGGTGCAGGTAGTCCA-3'
TIMP-1 (hum)	Forward	5'-TGTTGTTGCTGTGGCTGATAGC-3'
	Reverse	5'-TCTGGTGTCCCCACGAACTT-3'
TGFβ1 (hum)	Forward	5'-CTAATGGTGGAAACCCACAACG-3'
	Reverse	5'-TATCGCCAGGAATTGTTGCTG-3'
COL1A1 (hum)	Forward	5'-CGGTGTGACTCGTGCAGC-3'
	Reverse	5'-ACAGCCGCTTCACCTACAGC-3'
PAI-1 (hum)	Forward	5'-AGTGGACTTTTCAGAGGTGGA -3'
	Reverse	5'-GCCGTTGAAGTAGAGGGCATT-3'
SLC2A1 (hum)	Forward	5'-CAGTTTGGCTACAACACTGGAGT-3'
	Reverse	5'-ATAGCGGTGGACCCATGTCT-3'
HK2 (hum)	Forward	5'-GAGCCACCACTCACCCTACT-3'
	Reverse	5'-CCAGGCATTCGGCAATGTG-3'
PKM2 (hum)	Forward	5'-ATGTCGAAGCCCCATAGTGAA-3'
	Reverse	5'-TGGGTGGTGAATCAATGTCCA-3'
GPI (hum)	Forward	5'-CAAGGACCGCTTCAACCACTT-3'
	Reverse	5'-CCAGGATGGGTGTGTTTGACC-3'
LDHA (hum)	Forward	5'-ATGGCAACTCTAAAGGATCAGC-3'
	Reverse	5'-CCAACCCCAACAACTGTAATCT-3'
PFKM (hum)	Forward	5'-GGTGCCCGTGTCTTCTTTGT-3'
	Reverse	5'-AAGCATCATCGAAACGCTCTC-3'
PFKL (hum)	Forward	5'-GGAGAAGCTGCGCGAGGTTTAC-3'
	Reverse	5'-ATTGTGCCAGCATCTTCAGCATGAG-3'
ALDOA (hum)	Forward	5'-AGGCCATGCTTGCACTCAGAAGT-3'
	Reverse	5'-AGGGCCCAGGGCTTCAGCAGG-3'
PGK1 (hum)	Forward	5'-ATGTCGCTTTCTAACAAGCTGA-3'
	Reverse	5'-GCGGAGGTTCTCCAGCA-3'
PGAM1 (hum)	Forward	5'-GGAAACGTGTACTGATTGCAGCCC-3'
	Reverse	5'-TTCCATGGCTTTGCGCACCGTCT-3'
ENO1 (hum)	Forward	5'-GACTTGGCTGGCAACTCTG-3'
	Reverse	5'-GGTCATCGGGAGACTTGAA-3'
ENO2 (hum)	Forward	5'-TCATGGTGAGTCATCGCTCAGGAG-3'
	Reverse	5'-ATGTCCGGCAAAGCGAGCTTCATC-3'
MCT4 (hum)	Forward	5'-AGGTATCCTTGAGACGGTCAG-3'
	Reverse	5'-CAAGCAGGTTAGTGATGCCG-3'

Table S2. Primers for qRT-PCR (continued).

Gene	Sequence (5’-3’)	
β-actin (hum)	Forward	5’-CATGTACGTTGCTATCCAGGC-3’
	Reverse	5’-CTCCTTAATGTCACGCACGAT-3’
